# Family caregivers’ experiences of involvement in co-design research: A qualitative systematic review to develop involvement recommendations

**DOI:** 10.1016/j.ijnsa.2026.100730

**Published:** 2026-11-02

**Authors:** Maria Samuelsson, Camilla Skov, Emilie V. Bonde Hansen, Cristina Alfaro-Diaz

**Affiliations:** a Department of Care Science, Faculty of Health and Society, Malmö, Sweden; b Department of Oncology, Odense University Hospital, Odense, Denmark; c Department of Clinical Research, University of Southern Denmark, Denmark; d FaCe - Family focused healthcare research Center, University of Southern Denmark, Denmark; e Department of Nursing Care for Adult Patients, School of Nursing, Universidad de Navarra, Navarra, Spain; f IdiSNa, Navarra Institute for Health Research, Universidad de Navarra, Navarra, Spain

**Keywords:** Caregivers, Community-based participatory research, Family, Patient participation, Qualitative research, Research design, Systematic review

## Abstract

**Background:**

Co-design and participatory approaches that actively involve stakeholders in the research process have become increasingly prominent in healthcare and nursing research in recent decades. The growing number of publications has been accompanied by an increasing interest in mapping, collating, and synthesizing the existing evidence in the field. However, there has yet to be a systematic review that specifically explores the involvement of family caregivers in co-design research.

**Objective:**

This systematic review aims to synthesize evidence on family caregivers’ experiences of involvement in co-design research and to develop recommendations for involvement in future co-design projects.

**Information sources:**

PubMed, Cumulative Index to Nursing and Allied Health Literature, and PsycINFO.

**Methods:**

This qualitative systematic review employed a meta-aggregative approach, following the Joanna Briggs Institute’s guidelines, and is reported according to the Preferred Reporting Items for Systematic Reviews and Meta-Analyses. Systematic searches were conducted in November 2025 to identify primary studies that (1) describe the experiences of adult family caregivers of adult patients involved in co-design or participatory research; (2) employ a qualitative or mixed-methods design; and (3) are published in English, Spanish, Swedish, Norwegian, or Danish. Study selection was managed in Covidence, and titles, abstracts, and full-text records were screened independently by pairs of reviewers, with a third reviewer resolving discrepancies. The quality of the included studies was appraised using the Joanna Briggs Institute’s Critical Appraisal Checklist for Qualitative Research, and data were extracted using a customized template. Synthesized findings were generated from the extracted data, and their confidence was established using the ConQual approach. Lastly, recommendations for involvement were generated in collaboration with family caregivers and co-design researchers.

**Results:**

A total of 10,189 records were screened for relevance, of which 18 studies were included, describing experiences from 210 family caregivers involved in co-design research. The meta-aggregation generated five synthesized findings: motives for involvement, an open atmosphere, an authentic partnership, enablers and benefits of involvement. From these, six recommendations for involvement were developed. Overall, the quality of the studies was high, yet their ConQual assessment scores were low, largely owing to mixed stakeholder populations and insufficient reporting of researcher positionality and reflexivity.

**Conclusions:**

While many family caregiver experiences align with established principles of stakeholder engagement, unique motives and constraints shape their involvement. These findings and recommendations can support more meaningful and inclusive involvement of family caregivers. Future research should validate these findings and strengthen the representation of caregivers with high caregiving demands.

**Registration:**

PROSPERO CRD420251229190.

**What is already known**

•Co-design is associated with enhancing research relevance, quality, and uptake.

•There is an increased interest in stakeholder involvement in nursing research.

•Involving family caregivers in co-design remains challenging.

**What this paper adds**

•Family caregivers describe unique motives, benefits, and constraints.

•Meaningful involvement requires specific prerequisites beyond general principles.

•Family caregiver perspectives remain underrepresented in co-design research.

## 1 Background

Co-design and participatory approaches that actively involve stakeholders in the research process have become increasingly prominent in healthcare and nursing research in recent decades (Skivington et al., 2021, Bryant et al., 2022, Hall et al., 2021, Grindell et al., 2022). These approaches are grounded in principles such as democratic power sharing, relationship building, the inclusion of diverse skills and perspectives, respect for diverse forms of knowledge, reciprocity and collective ownership (Chastin et al., 2025, National Institute for Health and Care Research (NIHR), 2024, Grindell et al., 2022). Co-design research is commonly defined as a collaborative process in which researchers and stakeholders establish a partnership, and stakeholders take an active role in one or more stages of the research process—including formulating or prioritizing research questions, planning data collection, analyzing results, and disseminating findings (Eyles et al., 2016, Domecq et al., 2014, Molloy et al., 2019, Grindell et al., 2022). By sharing knowledge and decision-making, co-design enhances research relevance, quality, and uptake as well as improves recruitment, response, and retention rates. Consequently, conducting research *with* rather than *on* people has become an established principle of contemporary healthcare research ethics and practice (Skivington et al., 2021, Bryant et al., 2022, Hall et al., 2021, Grindell et al., 2022).

Family caregivers are a particularly important stakeholder group in co-design research. They possess experiential knowledge that extends beyond the direct experience of illness to include supporting patients in everyday life, navigating healthcare systems, coordinating care, communicating with professionals, and managing the practical, emotional, and relational consequences of illness (Alfaro-Diaz et al., 2026; Jagoda et al., 2024). Their perspectives may therefore identify needs, priorities, barriers, and opportunities that may not be fully captured by patients, healthcare professionals, or researchers alone (Jagoda et al., 2024). Meaningful involvement of family caregivers in co-design may consequently contribute to the development of research priorities, interventions, services, or care processes that are more relevant, acceptable, feasible, and responsive to the realities of patients and their families. However, the extent and nature of such involvement can vary considerably, ranging from consultation and feedback to more active, shared decision-making roles across different stages of the research process (Leslie et al., 2019, Rothausen et al., 2025, Slattery et al., 2020). Despite the growing emphasis on stakeholder engagement, driven by its potential to enhance the relevance, quality, and uptake of research findings, persistent challenges in engaging family caregivers in co-design processes have been highlighted (Rothausen et al., 2025; Alfaro-Diaz et al., 2026; Skivington et al., 2021). To date, no systematic review has specifically examined the involvement of family caregivers in co-design research, representing a significant gap in the literature that this review aims to address.

For this review, we adopt an inclusive definition of *family caregiver*, defining it by the relationship to the patient rather than by the level of involvement in care: thus, we use “family” and “family caregivers” interchangeably. This is corroborated by research highlighting conceptual overlaps and inconsistencies (Baudry et al., 2019) and by research emphasizing that family caregivers’ involvement may change across the illness trajectory (Christophe et al., 2022, Wang et al., 2018)*.* Given the specific needs of children (Inhestern et al., 2021), this review focuses on adult family caregivers of adult patients. Furthermore, due to the conceptual diversity of co-design terminology (Pearce et al., 2020, Grindell et al., 2022), *family caregivers’ involvement in co-design* refers to a structured collaborative process in which researchers and family caregivers work as partners and caregivers actively contribute to one or more stages or activities of the research process, including identifying or prioritising research questions, designing methods or interventions, interpreting findings, and disseminating results (Rothausen et al., 2025). This definition emphasises not simply whether family caregivers are present in a co-design process, but how they participate and the extent to which their experiential knowledge contributes to research decision-making.

A qualitative systematic review is particularly appropriate to address this gap because the available evidence is expected to encompass diverse experiences, perceptions, relationships, and contextual factors that cannot be adequately captured through quantitative measures alone (Noyes et al., 2019, Aromataris et al., 2024). Synthesising qualitative evidence can provide a deeper understanding of how family caregivers experience participation, why particular approaches facilitate or hinder their involvement, and what conditions may enable more equitable and meaningful partnerships between researchers and caregivers (Booth et al., 2019, Flemming et al., 2019). Such an understanding is necessary not only to describe the extent of caregiver involvement but also to identify underlying processes and mechanisms that may influence the quality and meaningfulness of participation across different co-design contexts (Flemming et al., 2019).

Therefore, this review seeks to bring together dispersed qualitative evidence on family caregivers’ involvement in co-design research (Alfaro-Diaz et al., 2026). Specifically, it will examine how caregivers are involved, the roles and contributions they undertake, their experiences and perceptions of participation, and the factors that facilitate or hinder meaningful involvement. Synthesizing these findings will provide a more comprehensive understanding of family caregivers’ participation and generate evidence-informed recommendations for involving them more effectively and equitably in future co-design research. Preliminary searches of PubMed, PsycINFO, Cumulative Index to Nursing and Allied Health Literature, the Cochrane Library, and PROSPERO identified no comparable published or ongoing systematic reviews.

### 1.1 Aim

This systematic review aims to synthesize evidence on family caregivers’ experiences of involvement in co-design research and to develop recommendations for involvement in future co-design projects.

### 1.2 Research questions

The main research question guiding this review is, What are family caregivers’ experiences of involvement in co-design research?

The specific research questions are as follows:

• What is known about and where are the gaps in understanding how family caregivers perceive their roles, contributions, and influence within co-design processes?
• What are the barriers and facilitators to family caregivers’ involvement in co-design research?
• What strategies or conditions promote a meaningful and sustained involvement of family caregivers in co-design research?

## 2 Methods

This qualitative systematic review employs the meta-aggregative approach to qualitative evidence synthesis, as outlined by the Joanna Briggs Institute (Aromataris et al., 2024). Meta-aggregation is a pragmatic method of qualitative evidence synthesis designed to produce findings that are directly applicable to practice and policy. Consistent with this approach, the review was conducted in three stages: (1) extraction of findings from included studies together with supporting illustrations and assessment of credibility, (2) aggregation of findings with similar meaning into categories, and (3) synthesis of categories into overarching synthesized findings representing shared experiences across studies. In keeping with the meta-aggregative methodology, findings were not re-interpreted beyond the meanings attributed by the original study authors but were aggregated to produce practice-relevant conclusions. The study was preceded by a protocol (Alfaro-Diaz et al., 2026), is registered in PROSPERO CRD420251229190, and is reported according to the Preferred Reporting Items for Systematic Reviews and Meta-Analyses guidelines (Page et al., 2021) (Supplemental file S1).

### 2.1 Search strategies

Following recommendations for meta-aggregation (Porritt, 2024), we used a revised Population, Phenomenon of Interest, and Context mnemonic to clarify the research question and guide the search strategy: The population was *family caregivers*, the phenomenon of interest was *experiences of involvement,* and the context was *co-design research.*

The search strategy was designed in collaboration with a research librarian and was informed by the Peer Review of Electronic Search Strategies Guideline Statement (McGowan et al., 2016) and Joanna Briggs Institute’s guidelines (Aromataris et al., 2024). To develop and refine the search strategy, preliminary scoping searches were conducted in EMBASE between September and October 2025. The final systematic literature search (presented in Supplementary File 2 S2) was subsequently conducted on November 28, 2025, in PubMed, CINAHL, and PsycINFO. The search terms were adapted to each database using controlled vocabulary (e.g., Medical Subject Headings) and Boolean operators. No restrictions were placed on the publication date. Moreover, backward citation searching and forward citation tracking were performed for all included studies, and the reference lists of relevant reviews identified during the screening were hand searched to identify additional studies.

### 2.2 Eligibility criteria

Studies were included if they were peer-reviewed primary studies published in English, Spanish, Swedish, Norwegian, or Danish. The selected languages were chosen pragmatically based on the language competencies within the review team, enabling direct assessment, extraction, and appraisal of studies without translation. Consistent with recommendations for systematic reviews, we sought to balance methodological rigor, feasibility, and resource considerations (Aromataris et al., 2024). Although language restrictions formed part of the eligibility criteria, no studies were excluded on the basis of language. Further, studies were included if they had a qualitative or mixed-methods design. Mixed-method studies were eligible if they included extractable qualitative data and a reported qualitative data collection method. Importantly, they had to describe the experiences of adult family caregivers of adult patients involved in co-design or participatory research at any stage of the research process (planning, conducting, or dissemination). Studies were excluded if no data from family caregivers could be separated from data from other stakeholders.

### 2.3 Study selection

Search results were exported to Covidence, a web-based software platform for systematic reviews, for screening and data management. Covidence facilitates study screening, full-text review, data extraction, and quality appraisal by enabling independent assessment and management of review records. Duplicates were removed automatically, and titles and abstracts were screened independently by pairs of reviewers, with a third reviewer resolving discrepancies. Full-text studies were then assessed independently by pairs of reviewers using the predefined eligibility criteria.

### 2.4 Quality appraisal

To assess the quality of included studies, we used the Joanna Briggs Institute’s Critical Appraisal Checklist for Qualitative Research (Lockwood et al., 2015). Quality appraisal was conducted by researchers with professional backgrounds in nursing and expertise in qualitative research and evidence synthesis. All studies that met the inclusion criteria were included regardless of methodological quality to capture the full range of available evidence. The appraisal tool was piloted on two studies and discussed within the review team to calibrate assessments and ensure a shared understanding of the appraisal criteria. Thereafter, each study was assessed independently by two reviewers. Disagreements were resolved through discussion until consensus was reached. See Supplemental file 3 S3 for the complete appraisal.

### 2.5 Data extraction

Data were extracted at a sub-theme level to ensure they adequately reflected family caregivers’ experiences. Studies were divided among the research team, which comprised researchers with expertise in qualitative methods, evidence synthesis, and family caregiving research. The included studies were divided among the research team, who extracted data using a customized template capturing

a) study characteristics and context, authors, year of publication, country, aim, setting/context, participants, co-design activities, and themes/main categories; and
b) findings on family caregivers’ self-reported experiences of involvement and supporting data extracts.

Following Lockwood et al. (2015), a “finding” was defined as a verbatim extract of the authors’ analytic interpretation of their data. To assess credibility (i.e., the level of support for each finding), we extracted comprehensive supporting data. In studies involving multiple stakeholders, we only extracted the findings that clearly referred to family caregivers or the findings that the authors stated were expressed by all stakeholder groups.

The template was piloted on two studies to ensure consistency. Findings were then extracted independently in pairs, with one reviewer (MS) involved in all extractions. Regular team meetings were held to resolve uncertainties and ensure consistent application of the extraction criteria. Findings and supporting illustrations were extracted as reported in the primary studies to preserve the original authors’ interpretations, resulting in variation in levels of abstraction across studies. Subsequently, MS reviewed all studies and extracted data for completeness and accuracy. Discrepancies were resolved through discussion until consensus was reached.

### 2.6 Assessing the credibility of findings

Following Joanna Briggs Institute’s guidelines (Lockwood et al., 2015), we assessed the credibility of the findings using the following categories:

**Unequivocal:** Findings accompanied by a contextually rich illustration that clearly supports the interpretation beyond reasonable doubt.

**Credible:** Findings accompanied by an illustration that supports the interpretation, although alternative interpretations remain possible.

**Unsupported:** Findings not supported by an illustration.

For studies including multiple stakeholder groups, only findings that could reasonably be attributed to family caregivers were extracted. Where attribution was unclear, findings were discussed within the review team before inclusion. Following recommendations (Lockwood et al., 2015), we excluded unsupported findings from synthesis. The findings, their level of support, and supporting data are presented in Supplemental file 4 S4.

### 2.7 Synthesis

In accordance with the Joanna Briggs Institute meta-aggregative approach, synthesis proceeded in two stages. First, all unequivocal and credible findings were inductively grouped into categories based on similarity of meaning. Categories were developed through repeated examination of findings and supporting illustrations, generated independently by the research team, and refined through discussion until consensus was reached. Second, categories were aggregated into synthesized findings, representing broader patterns across studies while maintaining fidelity to the original data. Consistent with meta-aggregation (Lockwood et al., 2015), the aim was not to generate new theory but to produce comprehensive and practically relevant synthesized findings grounded in the included studies.

### 2.8 Establishing confidence

Confidence in the synthesized findings was assessed using the ConQual approach (Munn et al., 2014), which evaluates credibility and dependability. All findings were initially rated as high confidence and downgraded when methodological or interpretive limitations were identified. Credibility was assessed based on the congruence between the findings and supporting data, categorized as unequivocal, credible, or unsupported. Dependability was assessed using the five methodological criteria specified in the ConQual approach (Q2, Q3, Q4, Q6, and Q7 of the Joanna Briggs Institute critical appraisal tool; marked with * in Supplemental file S3). In accordance with ConQual guidance, studies meeting four to five criteria were not downgraded, those meeting two to three criteria were downgraded one level, and those meeting zero to one criteria were downgraded two levels. Final ConQual scores were determined through discussion among the research team.

### 2.9 Developing recommendations

The research team developed statements based on the synthesized findings (e.g., “experiencing that involvement has an impact is important for meaningful involvement”). Each statement reflected a key aspect of family caregivers’ experiences identified in the meta-aggregation. The statements were presented to a healthcare advisory board of 12 family caregivers during a two-hour face-to-face consultation. Participants discussed the relevance of each statement, important considerations, strategies to support it, and examples from their own experiences. Notes were taken throughout the consultation.

Following the consultation, the research team reviewed the notes and integrated the family caregivers’ reflections into a set of preliminary recommendations. The recommendations were refined through discussion within the research team until consensus was reached. To assess their relevance, clarity, and applicability to co-design research, the preliminary recommendations were subsequently presented to three experienced co-design researchers in individual consultations. The co-design researchers were identified through snowball sampling within research networks and provided feedback on the wording, feasibility, and usability of the recommendations. This feedback informed the final wording of the recommendations.

### 2.10 Ethical considerations

Family caregivers and co-design researchers were consulted as experts. No personal, identifiable, or sensitive data were collected; therefore, ethical approval was not required under the Swedish Ethical Review Act (2003:460). The participants were informed about the study’s purpose and procedures, and they were informed that participation was voluntary. All the participants gave verbal consent.

### 2.11 Reflexivity

All members of the research team are nurses with expertise in family nursing and qualitative research. MS, CS and CAD have specific experience in co-design research involving family caregivers. To avoid unintentional bias, the team engaged in reflexive discussions to articulate their preunderstanding prior to initiating the review, which is reported in the protocol. Reflexivity was maintained throughout the review through continuous individual and group reflections.

## 3 Results

After removing duplicates, we screened 10,189 studies for relevance. A total of 18 studies were included in the review. The search and selection process is presented in Fig. 1.

**Fig. 1:**
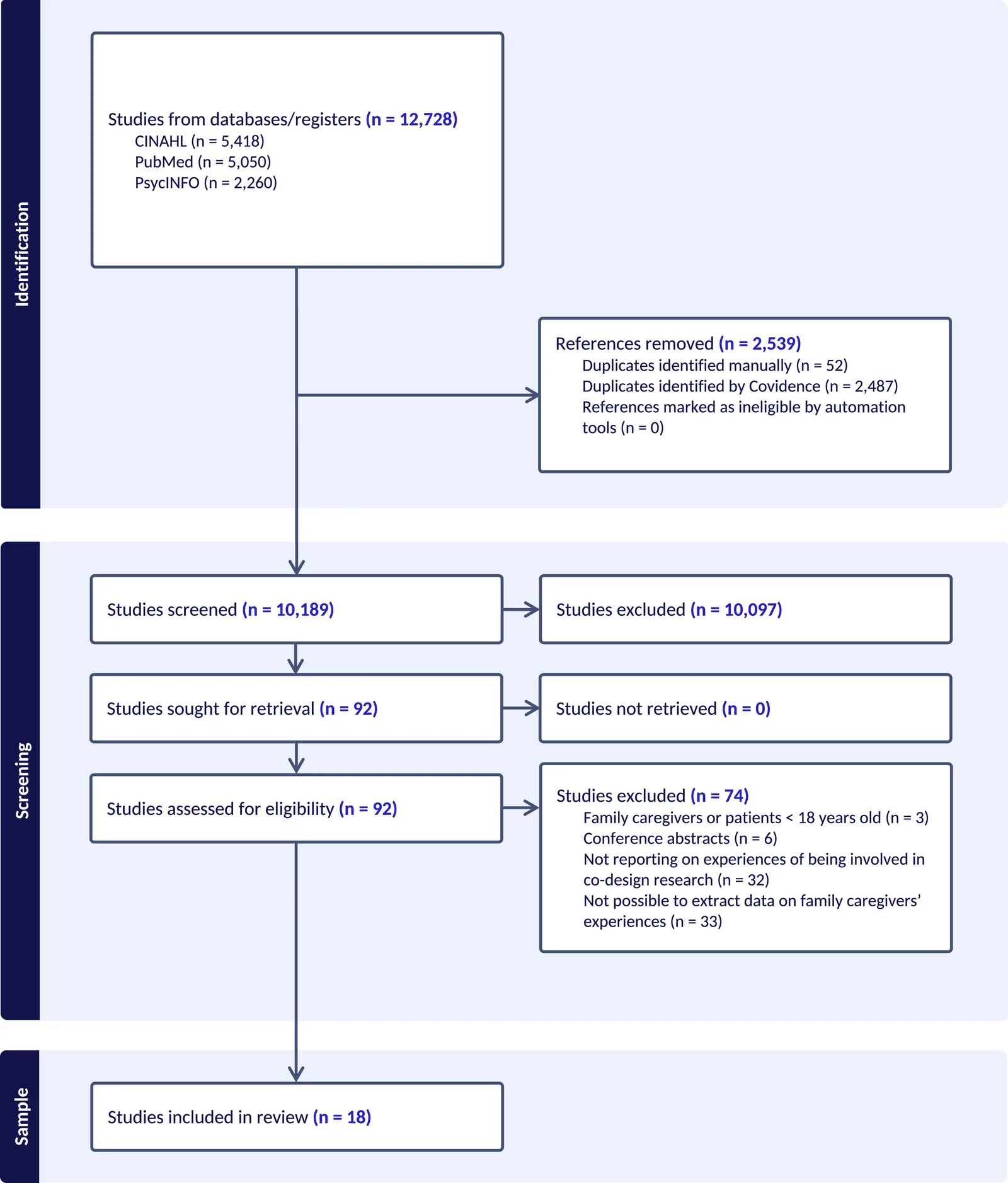
PRISMA flow diagram of the search and selection process.

### 3.1 Included studies

The included studies are presented in Table 1. They were conducted between 2015 and 2025. Family caregivers included in the review represented a range of countries, including Australia, Brazil, Canada, England, Ireland, Japan, Norway, Sweden, and the United States. The studies were conducted in the healthcare context of dementia (n = 6), intellectual disabilities (n = 3), stroke (n = 3), cancer (n = 1), kidney disease (n = 1), gerontology (n = 1), motor neuron disease (n = 1), mental health (n = 1), and multiple health conditions (n = 1). Of the 18 studies, 14 used a qualitative design, employing individual or focus group interviews for data collection, and 4 used a mixed or multi-method design, combining interviews with survey data. The number of participating family caregivers ranged from 2 to 51 (total n = 210). In four studies, only family caregivers participated (Coupe & Mathieson, 2020; Di Lorito et al., 2020; Lucchese et al., 2024; Nygaard et al., 2020); in the remaining studies, patients, healthcare professionals, and researchers also participated.

**Table 1:** Overview of the 18 included studies’ characteristics, family caregivers’ involvement in co-design, representation in the synthesized findings, main findings, and quality appraisal. .

**Author(s), Year, Country**	**Aim**	**Context**	**Design, Data collection, Analysis**	**Participants**	**Family caregivers’ involvement in co-design (activities)**	**Main findings**	**Quality appraisal (”yes” / all checklist items)**
Beighton et al., 2019, UK ^3,4,5^	To explore the perspectives and experiences of adults with intellectual disabilities and their parent carers regarding their public and participant involvement in a health research study over a three-year period	Intellectual disabilities	Qualitative study. Group interviews. Thematic analysis	Family caregivers (n = 4) and adults with a mild intellectual disability (n = 5)	Family caregivers were involved in the design, analysis, and dissemination phases through regular group meetings and discussions. They contributed to the selection and refinement of outcome measures, development of ideas for further analyses, interpretation of findings, co-presentation of results, and development of recommendations for future research and policy.	Ensuring authenticity of participation, working together, generating new outcome measures, disseminating findings, and involving caregivers in future research	10/10
Clarke et al., 2025, UK ^1,2,3,4,5^	To systematically explore the impact of Patient and Public Involvement and Engagement (PPIE) activities by addressing the facilitators and challenges of meaningful PPIE activities in research	Intellectual disability	Individual semi-structured interviews (audio-recorded and transcribed). Reflexive thematic analysis	Family carers (n = 5), adults with a mild intellectual disability (n = 2), researchers (n = 3), and PPIE facilitators (n = 2)	Family carers were involved as contributors in all research phases via advisory groups and co-production activities. They contributed to the development of the research proposal; informed research focus and outcome measures; reviewed study materials and procedures; supported recruitment; co-designed the intervention; and contributed to the interpretation and dissemination of findings, including through involvement in clinician training and study promotion.	Motivation for being a PPIE member, added value of PPIE input into research, logistics and practicalities of PPIE activities, and insights and reflections	10/10
Clarke et al., 2020, UK ^2,4,5^	To explore the facilitators of and barriers to using experience-based co-design (EBCD) and accelerated EBCD(AEBCD) in the development and implementation of interventions to increase activity opportunities for inpatient stroke survivors	Stroke rehabilitation research	Mixed method process evaluation. Semi-structured interviews. Integrative analysis	Family caregivers (n = 27), stroke survivors (n = 53), and staff (n = 76)	Family caregivers were involved at a **co-production level within the EBCD/AEBCD framework**, contributing from study inception (proposal development, Study Steering Committee membership) through co-design activities and feedback events to the interpretation and dissemination of findings. Their involvement included actively participating in co-design groups, reviewing study materials, and shaping service improvements.	Making sense of and engaging with EBCD/AEBCD over time; the facilitated EBCD/AEBCD process; patient and family member participation in EBCD/AEBCD; wider staff groups having less involvement in planning and actioning change; the challenges in implementing co-designed improvements; the influence of managerial authority; facilitating EBCD/AEBCD; and implementing and embedding co-designed changes into stroke unit practice	9/10
Coupe & Mathieson, 2019, UK ^3,4,5^	To explore the impact of patient and public involvement (PPI) on two health‐related doctoral research studies and identify how PPI could be used meaningfully at this level	Cancer research	Individual evaluation meetings (audio-recorded)	Family caregivers (n = 2)	Family caregivers were involved in targeted and embedded **consultations,** contributing to data collection, analysis, and dissemination as “Research Buddies.” They provided ongoing feedback on study progress, participated in data workshops (interpreting transcripts and developing themes), and informed implementation strategies and recommendations to ensure relevance for end-users.	Offering potential solutions when faced with recruitment problems, developing themes by drawing on experiences, and offering alternative explanations to themes identified in the data	6/10
Di Lorito et al., 2019, UK^2,3,4,5^	To propose a model for good practice in co-researching with carers of people with dementia	Dementia care research	Qualitative study. process evaluation. Reflective diaries	Family caregivers (n = 2)	Family caregivers were involved as co-researchers in all stages of the research cycle, including study design, development of materials, data collection, analysis, and dissemination. Their contributions included co-designing interview guides, conducting interviews, conducting independent data analysis, and co-authoring and presenting study findings.	Co-research yielded benefits for all those involved and on research outputs. There were practicalities and challenges that required extra resources to make the involvement of lay researchers meaningful and effective.	10/10
England et al., 2021, USA^1,2,4,5^	To understand the attitudes of homebound older adults and their caregivers toward research and participation as research advisors	Gerontology research of homebound older people	Individual semi-structured interviews (audio-recorded and transcribed). Descriptive thematic analysis	Family caregivers (n = 17) and homebound patients (n = 13)	Through the qualitative interviews, the family caregivers explored their attitudes toward research and their willingness to engage as research advisors. Their involvement was limited to the data collection phase, without active roles in study design, analysis, or dissemination.	Utility of research and relevance of research	7/10
Gutman et al., 2022, Australia^2.3,4,5^	To describe patient and caregiver experiences and perspectives of their previous involvement in research and to inform strategies to strengthen patient involvement in research	Chronic Kidney Disease research	Qualitative study. Individual semi-structured interviews. Thematic analysis	Family caregivers (n = 5) and patients (n = 18)	Family caregivers (with prior research involvement) contributed with insights to the priority setting, study design, recruitment, and dissemination. They also had key roles in the advisory and steering committees, workshops, document review, and participant recruitment.	Grappling with CKD diagnosis, bearing the responsibility for involvement, battling big agendas, seeing the person behind the patient, sensitivity to complexities of payment, and championing the patient voice	7/10
Harrison & Palmer, 2015, UK ^2,3,4,5^	To explore the experiences of patients and carers involved in PPI activities for stroke research.	Stroke research	Qualitative study.Individual semi-structured interviews. Thematic analysis	Family caregivers (n = 3) and patients (n = 8)	Family caregivers participated across research phases—spanning design, advisory work, analysis/interpretation, and dissemination—primarily through advisory groups, collaborative discussions with researchers, and review of study materials (e.g., lay summaries and information sheets).	Impact of PPI on the individual and the research process, credibility and expertise, level of involvement, and barriers and facilitators to PPI for stroke survivors and carers	7/10
Hemphill et al., 2019, USA^1,2,3,5^	To inform effective engagement practices and policies, we sought to un‐ derstand what motivates patients and caregivers to engage as partners on PCORI‐ funded research projects and how such engagement changed their lives	Patient‐Centered Outcomes Research Institute (PCORI) funded research projects focusing on a range of health conditions	Exploratory qualitative study. Open ended survey responses. Thematic analysis	Family caregivers (n = 51); patients (n = 116); representatives from patient, consumer, or caregiver advocacy organization (n = 41); and community‐based organizations (n = 35)	Family caregivers were involved as research partners on PCORI-projects contributing primarily through structured survey-based feedback (WE-ENACT). Their feedback informed study design, data collection tools, analytic approach, and interpretation of findings.	Motivations for engaging in research and changes in partners’ lives from engagement	7/10
Hudson et al., 2025, Australia^1,3,4,5^	To evaluate the co-design process from the perspectives of co-designers (individuals with aphasia, family members, and speech pathologists) and researchers (speech pathologists and technology developer) participating in EBCD workshops with respect to co-designer involvement, engagement, experiences, and personal outcomes	Stroke research	Mixed methods process evaluation within a convergent-parallel research design. Semi structured interviews (recorded and transcribed). The Framework Method and qualitative content analysis	Family caregivers (n = 4), individuals with aphasia (n = 4), and speech pathologists (n = 5)	Family caregivers were involved as co-designers across the EBCD co-design phase, participating in iterative design workshops, interviews, and evaluation activities alongside people with aphasia, clinicians, and researchers. They contributed with input throughout the collaborative design sessions and provided feedback on materials, app prototypes, and communication processes.	Involvement, engagement, experience, and personal outcomes	7/10
Lord et al., 2021, UK ^2,3,4,5^	To explore the experiences of people affected by dementia regarding their participation in the co-design of the New Interventions for Independence in Dementia Study (NIDUS)	Dementia care research	Mixed methods. Individual interviews (audio-recorded and transcribed). Thematic content analysis	Family caregivers (n = 10) and persons with dementia (n = 2) (participants in the qualitative data collection)	Family caregivers were involved in the co-design of the NIDUS-family intervention through co-design workshops and training sessions, and they provided iterative feedback on intervention development and materials, such as the structure and content of the theoretical model.	Creating the right atmosphere, participants influencing the outcome, having the right information, and unique challenges for people living with dementia	8/10
Lucchese et al., 2024, Canada, Ireland, Netherlands, UK, and Czech Republic^1,5^	To explore PPI engagement in health research with family carers to understand their interest in being involved in the Strategic Guiding Council within the Family Carer Decisions Support (FCDS) intervention	Dementia care research	Interpretative descriptive design.Individual semi-structured interviews (audio-recorded and transcribed). Thematic analysis	Family caregivers (n = 10)	Family caregivers were involved in the advisory and co-development phases of the FCDS intervention, engaging in structured discussions and in data collection for evaluation.	Engaging in reciprocal learning, using lived experience to support other family carers, and creating a collective momentum for advocacy and change	10/10
Masoud et al., 2021, USA^1,2,3,4,5^	To describe stakeholder experiences of the Stakeholder Advisory Council members engaged in the research process	Dementia care research	Qualitative study. Semi-structured individual interviews (audio-recorded and transcribed). Mixed inductive deductive analytic approach	Family caregivers (n = 4), persons with dementia (n = 2), and health care professionals (n = 5)	Family caregivers were engaged as members of a multi-stakeholder advisory council, contributing throughout the research process—including the priority setting, capacity-building activities, and interpretation of findings. They participated in meetings and co-learning activities (e.g., article reviews and webinars), and they contributed to data validation, analysis discussions, and manuscript development.	Accessibility, council infrastructure, values and environment, and benefits of involvement	9/10
Miah et al., 2020, UK Ireland, and Australia^2,3,4,5^	To evaluate the impact of PPI at each stage of the research cycle in a dementia research program	Dementia research	PPI monitoring forms and qualitative interviews (individual or dyadic interviews, audio-recorded and transcribed)	Family caregivers (n = 12), patients (n = 11), and researchers (n = 13)	Family caregivers were involved across all stages of the research cycle, contributing to priority setting, intervention and material design, data interpretation, and dissemination. They engaged in structured PPI activities, including co-developing and reviewing study materials, providing feedback on interventions, participating in the analysis (e.g., coding), and shaping strategies.	Impact on the research, impact on Research User Group members, and impact on researchers	7/10
Mousson et al., 2019, UK^1,2,4,5^	To explore the experiences of those who participate in, organize, and work with the Sheffield Motor Neurone Disorders Research Advisory Group (SMNDRAG)	Motor neuron disease research	Qualitative study. Semi-structured interviews (audio‐recorded and transcribed). Thematic analysis	Family caregivers (n = 3), patients (n = 2), researchers (n = 3), and association employees/volunteers (n = 5)	Family caregivers were involved as members of the SMNDRAG and contributed to the study design, material review, research prioritization, and dissemination. They participated in activities such as conducting protocol and document review, identifying research topics, advising on recruitment materials, and validating findings through manuscript review.	Motivations for joining/participating in the SMNDRAG, personal benefits of PPI, barriers and enablers to participating in PPI, barriers and enablers to effective and meaningful PPI, and perceived impact of PPI on research	10/10
Nygaard et al., 2020, Norway^1,2,3,4,5^	To describe the reflections of informal carers of people with dementia on the possibility of participating in the James Lind Alliance’s Priority Setting Partnership (PSP) process, for both themselves and the recipients of their care, and to explore barriers to and facilitators of their participation	Dementia research	Focus group interviews. Thematic analysis	Family caregivers (n = 36)	Family caregivers contributed to the early design phase via focus groups, providing experiential input on shaping PSP processes, including recruitment, communication strategies, and conditions for meaningful user involvement.	The creation of empowering teams where all voices are heard, the interaction of human factors, the PSP process and the environment, and the power of position and knowledge	7/10
Sutherland et al., 2025, UK ^2,3,4,5^	To describe the development of guidance on co-producing research with family carers of people with learning disabilities	Learning disabilities	Co-design. Working group meetings	Family caregivers (n = 10) and researchers (n = 7, including one researcher who is also a family carer)	Family caregivers were engaged as equal partners across all stages (design, conduct, analysis, and dissemination), participating in co-production meetings, mixed working groups, iterative document development, interviews, and co-authorship of guidance materials.	Work together as peers, make it meaningful, listen and keep an open mind, communicate clearly, pay appropriately, be sensitive, and be flexible	6/10
Yamaguchi et al., 2021, Japan^1,2,3,4,5^	To qualitatively analyze multiple stakeholders’ views on PPI, including potential concerns, barriers, and approaches	Community mental health research	Qualitative, Focus groups interviews. Data-driven analysis approach	Family caregivers (n = 5), patients (n = 6), service providers (n = 7), government staff (n = 5), and researchers (n = 14)	Family caregivers were providing input through focus group discussions, contributing to data generation on PPI perspectives. Their involvement was limited to the data collection phase, without engagement in study design, analysis, or dissemination.	Positive views and expectations regarding PPI, general concerns about PPI, specific issues regarding the implementation of PPI, and approaches to PPI implementation	8/10

The numbers 1–5 after the references refer to their representation in the synthesized findings: 1 = Motives for involvement, 2 = An open atmosphere, 3 = An authentic partnership, 4 = Enablers, and 5 = Benefits to support

### 3.2 Family caregivers’ experiences of involvement in co-design research

The meta-aggregation resulted in five synthesized findings: motives for involvement, an open atmosphere, an authentic partnership, enablers, and benefits of involvement. The ConQual score was “low” across the synthesized findings (Table 2). An overview of the meta-aggregation is presented in Fig. 2. The synthesized findings are discussed below and shown in Table 1 with reference to the included studies.

**Table 2:** ConQual assessment of the synthesized findings.

**Title:** Family caregivers’ experiences of involvement in co-design research: A qualitative systematic review to develop involvement recommendations			
**Bibliography**: Samuelsson, Skov, Bonde Hansen, (Alfaro-Diaz et al., 2026) International Journal of Nursing Studies Advances			
**Population**: Adult family caregivers (> 18 years) of adult patients (> 18 years)			
**Phenomenon of interest:** Experiences of involvement			
**Context:** Co-design research			
**Synthesized findings**	**Dependability**	**Credibility**	**ConQual score**
*Motives* for involvement include altruistic, caregiver, and personal incentives.	Downgrade 1 level*	Downgrade 1 level**	LOW
*An open atmosphere* is necessary for a true representation of family caregivers’ voices and emanates from a diversity of voices and a safe, collaborative environment.	Downgrade 1 level*	Downgrade 1 level**	LOW
*An authentic partnership* is necessary and achieved through a non-hierarchical relationship and visible impact.	Downgrade 1 level*	Downgrade 1 level**	LOW
*Enablers* are warranted and are provided through accessible information, flexibility, and tailored support.	Downgrade 1 level*	Downgrade 1 level**	LOW
*Benefits* of involvement include research benefits as well as personal and caregiving gains.	Downgrade 1 level*	Downgrade 1 level**	LOW

*Downgraded one level due to lack of reporting of Item ** Downgraded one level due to a mix of unequivocal and credible findings.

**Fig. 2:**
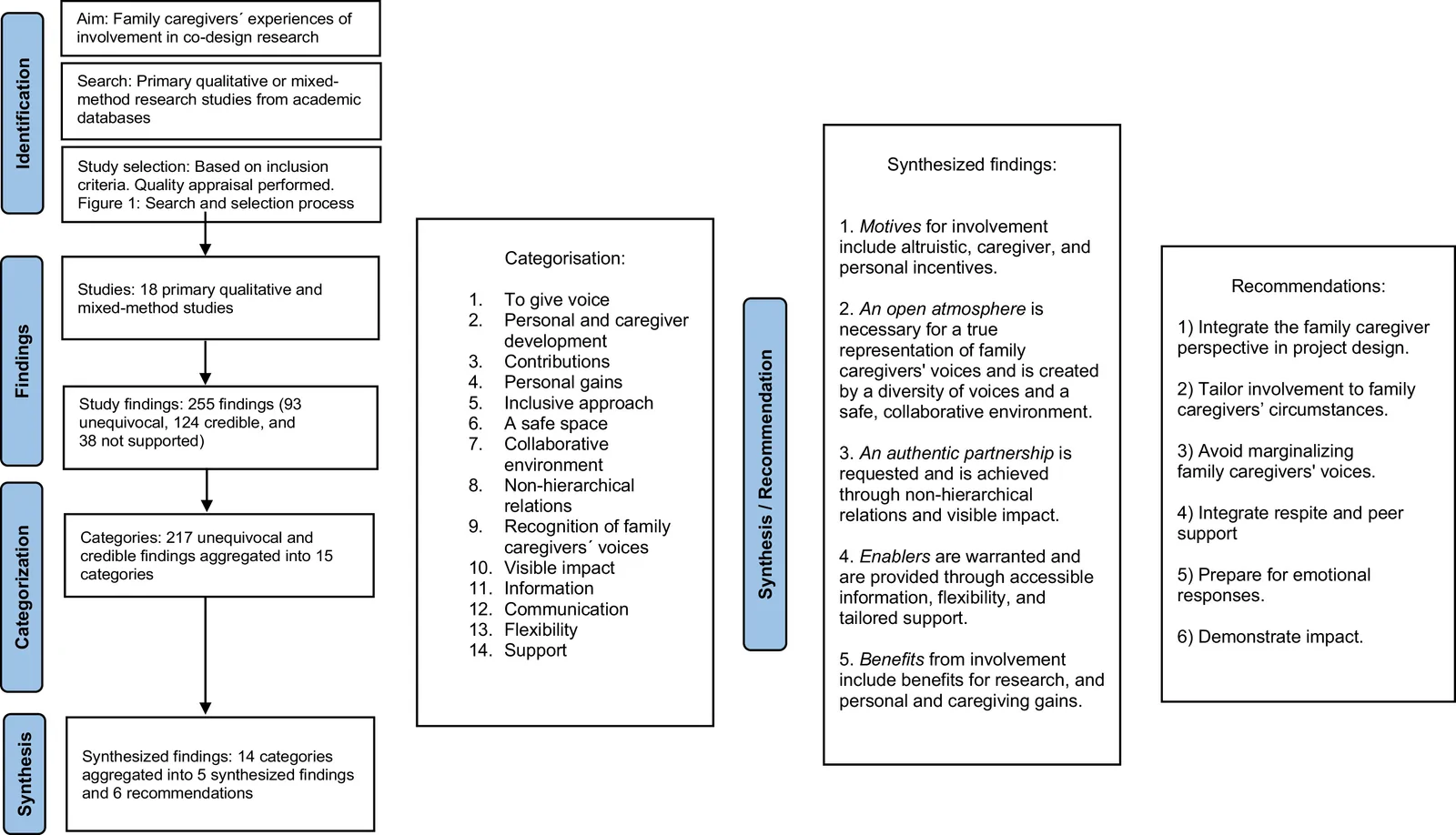
An overview of the meta-aggregation.

The synthesized findings collectively addressed the review questions. *Motives for involvement*, a*n authentic partnership*, and *benefits of involvement* illuminate how family caregivers perceived their roles, contributions, influence, and value within co-design processes. *An open atmosphere, an authentic partnership*, and *enablers* describe key barriers, facilitators, and conditions shaping involvement. Together, these findings identify the organisational, relational, and practical strategies required to support meaningful and sustained family caregiver involvement in co-design research. Fig. 3

**Fig. 3:**
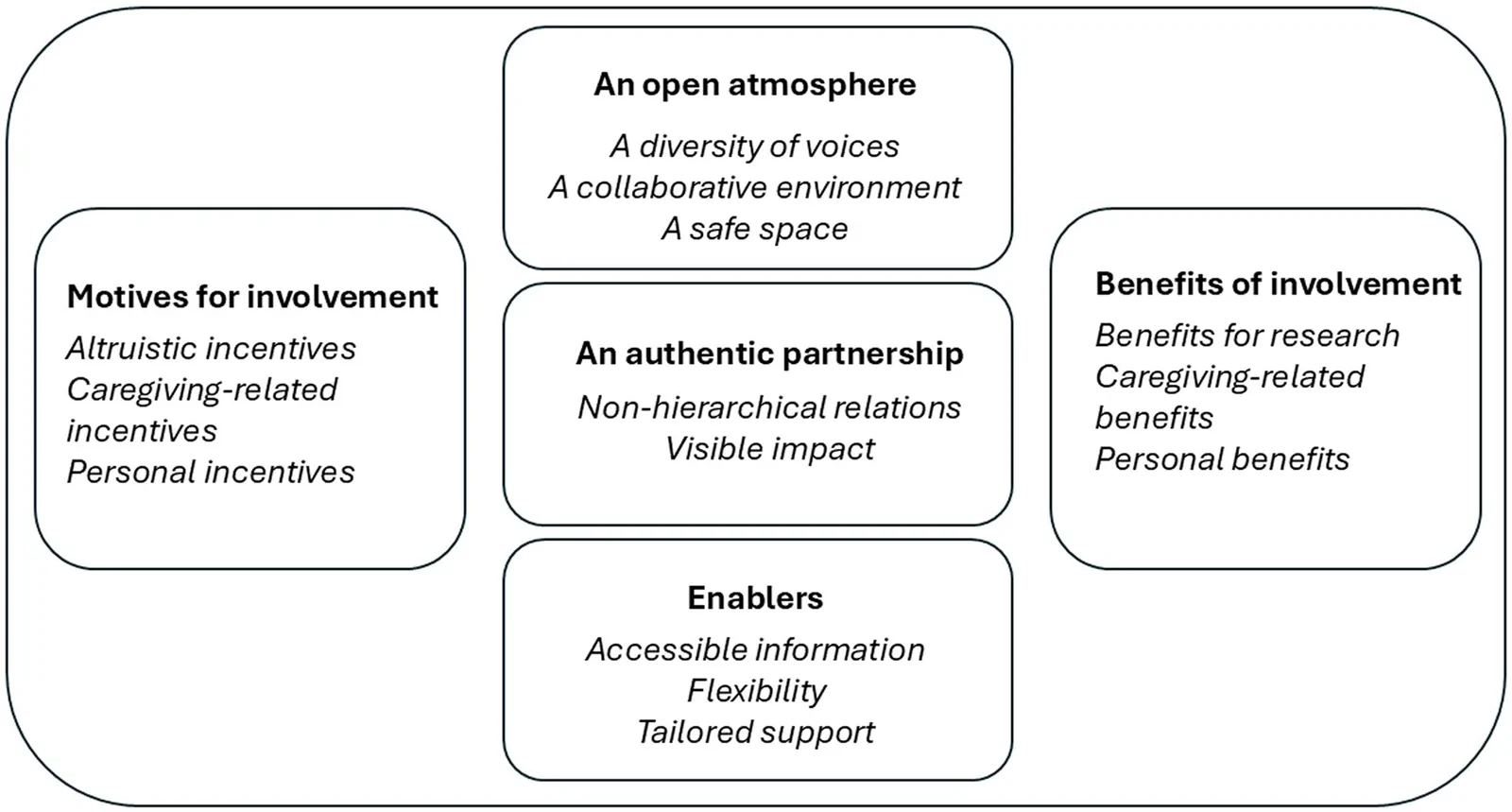
An overview of family caregivers’ experiences of being involved.

#### 3.2.1 Motives for involvement

*Motives for involvement* refers to the factors that motivate family caregivers to participate in co-design research. Family caregivers described altruistic, caregiving-related, and personal incentives for involvement, which shaped both their decision to participate and their expectations of the co-design process.

The altruistic incentives for participation included wanting to contribute their experiences to improve policy or practice for patients and caregivers in similar situations despite knowing the research could not benefit them personally (Hemphill et al., 2020, Clarke et al., 2025, Musson et al., 2019). Another altruistic motivation was sharing personal experiences to reduce feelings of shame in other family caregivers and to give voice to underrepresented groups, including family members with dementia and other marginalized patient and caregiver populations

(Nygaard et al., 2020). This act of “giving voice” was linked to a sense of responsibility to represent others, such as a family member with dementia. However, some caregivers felt that potential benefits for others were not sufficient and also wanted to perceive personal benefits from their involvement.

Caregiver-related incentives for participation included opportunities to learn from other family caregivers, healthcare professionals, and researchers in order to improve their caregiving skills. Personal incentives included meeting other family caregivers or turning painful experiences into something positive.

"I just feel I’ve got so much experience, so much knowledge, and so much sadness, I have to turn it into something positive. I need something good to come out of this. So that’s always been my driving force. I have to use it somehow". (Gutman et al., 2022)

#### 3.2.2 An open atmosphere

*An open atmosphere* refers to an inclusive and psychologically safe environment in which diverse perspectives are welcomed and family caregivers feel comfortable contributing their experiences.

Family caregivers emphasized that all perspectives—those of patients, family caregivers, and healthcare professionals—should be represented in research (Hemphill et al., 2020, Harrison and Palmer, 2015, Clarke et al., 2025). In addition, research should include voices from different caregiver groups (parents, siblings, partners) and sociodemographic populations. According to the family caregivers, representativeness is a matter of credibility, and its absence is a limitation:

“We need to get people who look like the people that we’re trying to reach: the Hispanics, the African Americans, the minorities, the people who are not as privileged. It can’t be the perception of this is a white person’s cure”. (Masoud et al., 2021)

Collating all perspectives and ensuring that everyone was heard could be complicated. Therefore, family caregivers stressed the importance of fostering a collaborative environment (Yamaguchi et al., 2022, Miah et al., 2020), particularly because family caregivers may be a lone voice in a research group and may find academic meetings daunting. Facilitators played a key role in fostering a warm, inclusive atmosphere, clarifying the purpose of participation, maintaining focus, and ensuring balanced contributions. This helped build family caregivers’ confidence to engage with researchers. A shared sense of purpose and strong group connectedness further supported collaboration and helped the group navigate disagreements:

“Despite disagreements, members believed the group worked well because they had ‘the same aim’”. (Musson et al., 2019)

A key component for family caregivers was an inclusive environment in which they felt safe sharing their opinions and perspectives:

“The researchers made us feel very comfortable and we were in a safe place and we could talk freely”. (Lord et al., 2022)

To support a sense of safety, researchers and family caregivers welcomed others’ contributions with openness and curiosity, particularly critical perspectives. Family caregivers also emphasized that sharing experiences could involve discussing deeply personal emotions, such as grief, distress, shame, and guilt, which could be both rewarding and challenging (Nygaard et al., 2020). Therefore, researchers must be mindful of family caregivers’ well-being throughout their collaboration and provide adequate support. This includes emphasizing that family caregivers may share as much or as little as they wish, interacting with family caregivers empathetically, and offering debriefs or follow-up support.

#### 3.2.3 An authentic partnership

*An authentic partnership* refers to relationships in which family caregivers are recognised as equal contributors whose views can meaningfully influence decisions and outcomes.

Equal, collaborative relations among participants were a key factor in an authentic partnership (Sutherland et al., 2025, Hudson et al., 2025, Coupe and Mathieson, 2020). Family caregivers perceived the researchers’ typical approach to engagement as hierarchical and predatory: Researchers enter communities to extract information without giving back. Therefore, family caregivers aspired to have a non-hierarchical collaborative/participatory model of involvement rather than a consultative model:

"Equality is very important. I think it is crucial to have a proper discussion in a partnership". (Yamaguchi et al., 2022)

Moreover, while embedded in co-research teams, some family members felt valued and integrated, whereas others described power imbalances among team members and a chasm between patients, family caregivers, and clinicians/researchers, which hindered family caregivers’ contributions (Nygaard et al., 2020, Gutman et al., 2022, Di Lorito et al., 2020). Some family caregivers also described difficulties in defining their role, feeling “on a much lower level” than other members of the research team (Gutman et al., 2022). These experiences may reflect researchers’ use of complex jargon, caregivers feeling that their contributions were undervalued, and uncertainty about what meaningful research collaboration involves. As a result, some caregivers were hesitant to engage in co-research tasks and called for broader cultural change within academia to better recognize the value of lay researchers (Di Lorito et al., 2020). Conversely, when researchers used appropriate language, made genuine efforts to understand family caregivers’ experiences and views, recognized their role and contribution, and were open to questioning, they were able to establish a non-hierarchical relationship that fostered authentic partnership:

"I thought that not only our voices were heard but they were listened to". (Clarke et al., 2025)

Further, family caregivers emphasized that an authentic partnership meant having an impact. Otherwise, their involvement would be just for show. They wanted those in policy and practice to hear their voices. However, they were aware that their involvement was crucial in enabling the researchers to secure funding and were apprehensive of tokenistic involvement—of being a tick in a box and an alibi for funding (Beighton et al., 2019). The feeling of tokenistic involvement was prompted by situations such as a lack of opportunities for family caregivers to provide feedback and researchers presenting ready-made solutions and definitive answers or appearing to have made decisions in advance.

"I got the impression that I was telling them probably useful information but too late. I think they’d already made up their mind what they wanted to do and it was too late to take on-board any further even if it might have been useful". (Lord et al., 2022)

Therefore, family caregivers suggested involvement from the early stages of research projects to shape key decisions and to meaningfully influence the course of research. When their contributions were recognized as valid and important and there was a clear plan to incorporate them, their involvement felt genuine:

"I genuinely felt, and I’ve said this to various people, but this wasn’t just a tick box exercise, ooh yes, I’ve consulted carers, it was a genuine . . . let’s see how you can get involved and I’d like to incorporate your ideas in it, so it did feel like genuine involvement which was great". (Beighton et al., 2019)

In addition, family caregivers appreciated researchers providing them with feedback about how their contributions were carried forward and used, and when they did not receive such information, they requested it:

"Often, you hear nothing afterward. Complete silence. The information you’ve given is just stored in a database or something. Involvement and follow-up are very important. One must know the what, how, and why". (Nygaard et al., 2020)

#### 3.2.4 Enablers

*Enablers* refer to the practical, informational, and organisational conditions that support family caregivers’ ability to participate in co-design activities. Table 1 for included references.

In particular, family caregivers needed information that outlined, explained, and guided them through the co-design research process (Di Lorito et al., 2020, Musson et al., 2019, Clarke et al., 2021). They needed to understand the purpose of their involvement and their role in the process, namely what was expected of them, when, and how. Their lack of awareness and understanding of co-design and uncertainty about the contribution they could make were the main barrier to involvement:

"Not knowing exactly what it involves… and feeling that they have nothing to offer". (Musson et al., 2019)

Furthermore, clarity on what could be changed and what could not was crucial. For instance, caregivers found unexpected limitations, such as those related to ethical approval or healthcare systems, disappointing or frustrating. Some family caregivers also felt that having less information than the researchers in the team could impede meaningful engagement:

"I think sometimes the research, you know, it was slightly muddled… [we] sometimes get a bit confused about how the manual was supposed to work, at what…which point it was going to be used when…You know what I mean? Not necessarily something that we would prepare for because it was all totally clear to them. I suppose that’s partly the process". (Lord et al., 2022)

In addition, family caregivers requested a project overview, especially in large research projects. They expressed feeling excluded during prolonged intervals between research activities, indicating a need for regular updates:

"I didn’t really know what was happening… You still feel some involvement in it, but it’s, sort of, been…I feel, as you say, a bit distanced from it". (Lord et al., 2022)

When receiving this information, family caregivers preferred to receive it in an accessible structure and format through convenient channels continuously throughout the project period (Clarke et al., 2025, Masoud et al., 2021, Sutherland et al., 2025). When researchers provided training and a simple and inclusive presentation of practices, free from technical jargon, they increased family caregivers’ confidence in their abilities to make meaningful contributions that would have an impact on the research. However, some family caregivers felt they only needed basic training; in particular, those who placed the greatest value on their experiential knowledge did not want training and felt that it might detract from their lay role.

Importantly, caregiving responsibilities affected family caregivers’ prerequisites for involvement, prompting their need for facilitation (Nygaard et al., 2020, Gutman et al., 2022, Eaton England et al., 2021). Some family caregivers felt they could not leave the person in their care unattended, were already tired from caregiving, had no spare time, or felt that their involvement would disturb their everyday routine. For instance, many of them were “time-poor” and often had to respond to unexpected, last-minute demands:

"And you must facilitate participation. That was a big problem, I could barely leave my husband for long enough to get to the mailbox". (Nygaard et al., 2020)

The illness of the family member receiving care was another factor:

"Not tiring for me, it’s tiring for such as Berol or [name of other stroke survivor], they get tired after 2 hours". (Harrison and Palmer, 2015)

That is, family caregivers felt that researchers need to understand the specific impact of a diagnosis on the patient, the family caregiver, and their everyday lives and adapt research processes accordingly. Such adaptations were considered important to support the meaningful involvement of family caregivers as stakeholders in their own right, rather than merely as facilitators of patient participation. Consequently, family caregivers needed flexibility and tailored support to facilitate their involvement (Sutherland et al., 2025, Harrison and Palmer, 2015, Clarke et al., 2025). Practical facilitators included holding meetings in disability-friendly environments, providing transport, and offering digital meetings and flexible time schedules. Further, researchers could offer family caregivers different input methods, catch-up sessions, and varying degrees of involvement based on their availability. Different family caregivers have different needs, and researchers should be willing to explore reasonable adaptations to enable them to contribute.

#### 3.2.5 Benefits of involvement

*Benefits of involvement* refers to the perceived positive outcomes of participating in co-design research. Family caregivers described benefits related to the research, their caregiving role, and their personal lives, highlighting how involvement could generate value for both the research process and the individuals involved. Table 1 for included references.

The experienced benefits for research included incorporating a perspective that highlighted the lived experiences of patients and family caregivers, thereby contributing valuable knowledge and challenging researchers assumptions (Di Lorito et al., 2020, Miah et al., 2020, Eaton England et al., 2021). Furthermore, the lay perspective helped researchers refine the research questions, assess feasibility, and shape realistic expectations of what interventions could achieve. Additionally, family caregivers and researchers perceived a synergistic effect of different perspectives working together.

Involvement also helped family caregivers learn more about patient diagnosis, care processes, and the healthcare system, thereby advancing their caregiving skills (Lucchese et al., 2024). On a personal level, family caregivers found gaining insight into research empowering and felt that collaborating with healthcare professionals reduced barriers to meaningful dialogue. They valued being listened to, making a contribution, transforming difficult experiences into something positive, and building supportive relationships with peers. Some also viewed involvement as a welcome respite from caregiving:

"But also it’s the getting away from it, you know, and having a piece of cake and a cup of tea". (Harrison and Palmer, 2015)

## 4 Recommendations

The recommendations below were derived from the synthesized findings and further developed through consultations with a family caregiver advisory board and experienced co-design researchers.

**Integrate the family caregiver perspective in project design:** Involve family caregivers from the intended context early in the design process to ensure their perspectives inform key decisions.

**Tailor involvement to family caregivers’ circumstances:** Explore support needs with family caregivers early in the project and provide flexible, accessible, and tailored opportunities for involvement that accommodate caregiving responsibilities and everyday constraints.

**Avoid marginalizing family caregivers’ voices:** (A) Create a safe environment for sharing perspectives through clear information, guidance, and team-building activities. Use the facilitator role to reduce power imbalances by emphasizing shared goals and the value of diverse expertise. (B) Support family caregivers in recognizing the value of their unique expertise. (C) Encourage caregivers’ perspectives beyond their role as proxies for the patient. (D) Avoid positioning caregivers as “lone voices” in mixed groups and consider homogeneous or individual sessions.

**Integrate respite and peer support:** Offer activities that provide personal benefit to caregivers, such as activities that create opportunities for informal peer interaction (e.g., homogeneous coffee breaks).

**Prepare for emotional responses:** Anticipate that involvement may elicit sensitive emotions and ensure sufficient time, emotional support, and appropriate safeguards. Sensitive emotions, per se, do not lead to a negative framing/perception of the workshop but can be an opportunity to “finally talk about these things even if it is difficult.”

**Demonstrate impact:** Provide ongoing feedback on how caregivers’ contributions are acknowledged and used in the co-design project.

## 5 Discussion

This systematic review identified five synthesized findings on family caregivers’ involvement in co-design research: motives for involvement, an open atmosphere, authentic partnership, enablers, and benefits. Based on these findings, involvement recommendations were co-developed with family caregivers and co-design researchers. Overall, meaningful involvement appears to depend on caregivers’ motivations, the quality of partnerships, and organisational conditions that support engagement.

Our findings both confirm and extend existing knowledge on stakeholder involvement in co-design. Consistent with previous research on patient and public involvement and co-design (e.g. Blackwell et al., 2017; Peters et al., 2024; Vargas et al., 2026), family caregivers emphasised the importance of non-tokenistic involvement, collaborative relationships, psychological safety, and adequate support for participation. These factors have been widely recognised as prerequisites for meaningful stakeholder engagement. However, by synthesising evidence across caregiving contexts, the review moves beyond generic principles of stakeholder engagement and identifies caregiving responsibilities as a central contextual factor shaping participation. Our findings suggest that family caregivers experience involvement through a distinct caregiving lens. Unlike many other stakeholders, their opportunities to participate were strongly shaped by ongoing caregiving responsibilities, the health status of the care recipient, and competing demands on their time and emotional resources (e.g,. Sutherland et al., 2025, Harrison and Palmer, 2015, Clarke et al., 2025). Meaningful involvement therefore depended not only on relational and organisational conditions, but also on whether participation could be accommodated within everyday caregiving realities. Importantly, our findings suggest that family caregivers with high caregiving demands may face particular challenges in participating in co-design activities. Without flexible and tailored approaches to involvement, there is a risk that the perspectives of caregivers with the greatest caregiving responsibilities remain underrepresented.

This distinction is important because co-design frameworks often focus on power-sharing and collaboration (Blackwell et al., 2017; Peters et al., 2024; Vargas et al., 2026), while paying less attention to the practical constraints associated with caregiving. Our findings suggest that involvement strategies that may be sufficient for other stakeholder groups are not necessarily sufficient for family caregivers. Flexible participation options, tailored support, regular communication, and recognition of caregiving demands may therefore be particularly important when seeking to involve family caregivers in co-design research. This finding suggests that existing co-design frameworks may benefit from a more explicit consideration of caregiving circumstances when planning, implementing, and evaluating involvement activities.

A particularly noteworthy finding was that caregiver-specific experiences were most evident in studies focusing exclusively on family caregivers (e.g., Nygaard et al., 2020), whereas they were less visible in studies combining multiple stakeholder groups. For example, in one mixed-stakeholder study, caregiver-specific experiences were reported only within a category labelled “less commonly reported experiences” (Hemphill et al., 2020). This raises the possibility that family caregivers’ perspectives may become diluted when analysed alongside those of patients, clinicians, and researchers. While co-design aims to bring together multiple perspectives, there is a risk that family caregivers are positioned primarily as supporters of patients rather than as stakeholders with their own experiences and support needs. Our findings therefore suggest that researchers should critically consider how family caregivers’ voices are captured, analysed, and represented throughout co-design processes. Moreover, researchers more frequently reported strategies aimed at facilitating patient participation, such as disability-friendly environments, transportation support, and the inclusion of family caregivers, whereas strategies specifically designed to support family caregivers’ involvement were less commonly described. This observation aligns with previous research suggesting that family caregivers are often positioned in the shadow of the patient (Molassiotis and Wang, 2022; Samuelsson et al., 2022), highlighting the need for deliberate strategies that bring caregiver perspectives to the foreground.

Collectively, these findings suggest that family caregivers should not merely be viewed as another stakeholder group within co-design research. Rather, their involvement is shaped by the unique realities of caregiving, requiring approaches that acknowledge both their experiential expertise and the practical constraints associated with caregiving responsibilities. By synthesising family caregivers’ experiences across diverse co-design contexts, this review contributes a caregiver-centred perspective that has been largely absent from the broader co-design literature.

The ConQual assessment resulted in low confidence ratings despite generally high methodological quality. This was mainly due to mixed stakeholder samples, which limited the depth of family caregivers’ perspectives, and insufficient reporting of researchers’ positionality and reflexivity. While the findings are supported by the available evidence, further high-quality studies focusing on homogeneous caregiver groups and transparent reporting of researchers’ roles and reflexivity are needed. Such transparency is essential for rigorous qualitative research, particularly in co-design studies.

A total of 210 family caregivers participated across the included studies; however, only four studies specifically focused on family caregivers (Coupe & Mathieson, 2020; Di Lorito et al., 2020; Lucchese et al., 2024; Nygaard et al., 2020). As noted, most studies included multiple stakeholders (Table 1), and two studies included only two participants (Coupe & Mathieson, 2020; Di Lorito et al., 2020). Consequently, the themes presented in the reviewed literature largely reflect shared experiences across stakeholders rather than experiences unique to family caregivers. To address this, we extracted data at the sub-theme level, foregrounding family caregivers’ voices in particular.

### 5.1 Strengths and limitations

A key strength of this review is its adherence to established guidelines, transparent reporting, and a comprehensive search strategy developed with an experienced research librarian. The meta-aggregative approach enabled the synthesis of qualitative findings into actionable recommendations, which were further refined through input from a family caregiver advisory board and co-design researchers.

Some limitations should be acknowledged. Despite a comprehensive search strategy, relevant studies may have been missed. In addition, only studies published in English, Spanish, Swedish, Norwegian, and Danish were included. Consequently, evidence published in other languages, such as French, German, Mandarin, or Japanese, may have been overlooked, potentially introducing language bias and limiting the representation of diverse cultural and healthcare contexts. Although the included studies represented a range of countries and settings, some cross-cultural perspectives may not have been captured. Furthermore, despite independent screening by pairs of reviewers, the large number of retrieved records may have resulted in some relevant studies being missed.

To ensure good quality, we applied the Joanna Briggs Institute’s Critical Appraisal Checklist for Qualitative Research. However, several studies were not “traditional” qualitative studies (Table 1), which complicated their quality assessment. Based on our experiences with this review and a previous scoping review of the co-design of family interventions in cancer care (Rothausen et al., 2025), we look forward to quality assessment guidelines for this research field.

Another limitation concerns the review’s intentionally broad focus on family caregivers’ experiences of involvement in co-design regardless of context. Such experiences may be influenced by factors such as illness trajectories, treatment modalities, and the availability of formal and informal support systems, which vary across contexts (Christophe et al., 2022, Molassiotis and Wang, 2022). Consequently, the synthesized findings may not be directly transferable to all family caregiving contexts. Nevertheless, given the existing literature indicating shared and recurring experiences among family caregivers (Christophe et al., 2022, Molassiotis and Wang, 2022), this review yields insights that can inform general recommendations we believe are adaptable to specific populations and local care contexts.

Notably, the recommendations generated in this review are based on the experiences of family caregivers who were able to participate in co-design activities. Consequently, caregivers who are most heavily involved in daily care and, therefore, less able to engage in such initiatives may be underrepresented. We sought to mitigate this limitation by involving family caregiver representatives and co-design researchers in the development of the recommendations. Nevertheless, this remains a critical issue, and we strongly recommend that future research explicitly examine how caregivers with high caregiving demands can be included and adequately represented.

## 6 Conclusions

The meta-aggregation of the available evidence on family caregivers’ experiences of involvement in co-design research generated five synthesized findings: motives for involvement, an open atmosphere, an authentic partnership, enablers, and benefits of involvement. Based on these findings, recommendations for involvement in co-design research were developed in collaboration with family caregivers and co-design researchers. Overall, the reviewed studies were of high quality, yet their ConQual assessment scores were low, largely due to the predominance of mixed stakeholder populations and the insufficient reporting of researcher positionality and reflexivity, highlighting the need for improved methodologies in future research.

While many experiences identified in this review align with established principles of stakeholder engagement, family caregivers also described unique motives and constraints, such as participation as respite or peer support, difficulties leaving the person they care for unattended, and limitations related to that person’s health status. These findings suggest that meaningful involvement requires not only openness and partnership but also practical support, including flexible participation options, accessible communication, role clarification, tailored accommodations, and recognition of caregivers’ competing responsibilities. Researchers should actively facilitate family caregivers’ influence throughout co-design processes and remain attentive to the risk that their voices may be overshadowed in mixed-stakeholder settings.

The findings and recommendations can inform future co-design research involving family caregivers. Further research is needed to explore caregiver-specific populations, identify ways to include caregivers with high caregiving demands, and evaluate strategies that support meaningful involvement and improve co-design outcomes. Greater attention to researcher positionality, reflexivity, and transparent reporting is also needed to strengthen the trustworthiness of future research.

## Funding

This research did not receive any specific grant from funding agencies in the public, commercial, or not-for-profit sectors.

## CRediT authorship contribution statement

**Maria Samuelsson:** Writing – review & editing, Writing – original draft, Project administration, Methodology, Formal analysis, Conceptualization. **Camilla Skov:** Writing – review & editing, Validation, Methodology, Formal analysis, Conceptualization. **Emilie V. Bonde Hansen:** Writing – review & editing, Methodology, Conceptualization. **Cristina Alfaro-Diaz:** Writing – review & editing, Methodology, Formal analysis, Conceptualization.

## Declaration of competing interest

The authors declare that they have no known competing financial interests or personal relationships that could have appeared to influence the work reported in this paper.
