# Supplementary material for: Family caregivers’ experiences of involvement in co-design research: A qualitative systematic review to develop involvement recommendations
Source: Int J Nurs Stud Adv. 2026 Nov 2;11:100730. doi: 10.1016/j.ijnsa.2026.100730 (PMC13770637; doi:10.1016/j.ijnsa.2026.100730)
Supplement: Supplementary file 1 [file mmc1.docx]

# Family caregivers' experiences of being involved in co-design research – Search strategies

## PubMed (NLM) date searched 251128

| **Caregivers**  (((((((((((((("Caregivers"[Mesh]) OR ("Family"[Mesh])) OR (caregivers[Title/Abstract])) OR (caregiver[Title/Abstract])) OR (family[Title/Abstract])) OR (families[Title/Abstract])) OR (mothers[Title/Abstract])) OR (mother[Title/Abstract])) OR (fathers[Title/Abstract])) OR (father[Title/Abstract])) OR (carers[Title/Abstract])) OR (carer[Title/Abstract]))) OR (relatives[Title/Abstract])) OR (relative[Title/Abstract])) OR (next of kin[Title/Abstract]) | 1,258,851 |
| --- | --- |
| **Participatory research**  (((((("Community-Based Participatory Research"[Mesh]) OR (Participatory Research[Title/Abstract])) OR (citizen science[Title/Abstract])) OR (co-researcher[Title/Abstract])) OR (coresearcher[Title/Abstract])) OR (co-researchers[Title/Abstract])) OR (coresearchers[Title/Abstract]) | 15,149 |
| **Co-design**  (((((((((((co-design*[Title/Abstract]) OR (codesign*[Title/Abstract])) OR (participa* design*[Title/Abstract])) OR (co-creat*[Title/Abstract])) OR (cocreat*[Title/Abstract])) OR (co-produc*[Title/Abstract])) OR (coproduc*[Title/Abstract])) OR ((stakehold*[Title/Abstract]) AND (((engag*[Title/Abstract]) OR (involv*[Title/Abstract])) OR (collaborat[Title/Abstract])))) OR ((consumer*[Title/Abstract]) AND (((engag*'[Title/Abstract]) OR (involv*[Title/Abstract])) OR (collaborat*[Title/Abstract])))) OR ((participant[Title/Abstract]) AND (((engag*[Title/Abstract]) OR (involv*[Title/Abstract])) OR (collaborat*[Title/Abstract])))) OR (patient and public involv*[Title/Abstract])) OR (public contribut*[Title/Abstract]) | 101,885 |
| **Research**  ((((((("Research"[Mesh]) OR ("Clinical Trials as Topic"[Mesh])) OR ("Research Personnel"[Mesh])) OR (research[Title/Abstract])) OR (clinical trial*[Title/Abstract])) OR (clinical study[Title/Abstract])) OR (clinical studies[Title/Abstract])) OR (science[Title/Abstract]) | 4,245,293 |
| **Co-design AND research**  ((((((((((((co-design*[Title/Abstract]) OR (codesign*[Title/Abstract])) OR (participa* design*[Title/Abstract])) OR (co-creat*[Title/Abstract])) OR (cocreat*[Title/Abstract])) OR (co-produc*[Title/Abstract])) OR (coproduc*[Title/Abstract])) OR ((stakehold*[Title/Abstract]) AND (((engag*[Title/Abstract]) OR (involv*[Title/Abstract])) OR (collaborat[Title/Abstract])))) OR ((consumer*[Title/Abstract]) AND (((engag*'[Title/Abstract]) OR (involv*[Title/Abstract])) OR (collaborat*[Title/Abstract])))) OR ((participant[Title/Abstract]) AND (((engag*[Title/Abstract]) OR (involv*[Title/Abstract])) OR (collaborat*[Title/Abstract])))) OR (patient and public involv*[Title/Abstract])) OR (public contribut*[Title/Abstract])) AND (((((((("Research"[Mesh]) OR ("Clinical Trials as Topic"[Mesh])) OR ("Research Personnel"[Mesh])) OR (research[Title/Abstract])) OR (clinical trial*[Title/Abstract])) OR (clinical study[Title/Abstract])) OR (clinical studies[Title/Abstract])) OR (science[Title/Abstract])) | 52,627 |
| **Participatory research OR (co-design AND research)**  ((((((("Community-Based Participatory Research"[Mesh]) OR (Participatory Research[Title/Abstract])) OR (citizen science[Title/Abstract])) OR (co-researcher[Title/Abstract])) OR (coresearcher[Title/Abstract])) OR (co-researchers[Title/Abstract])) OR (coresearchers[Title/Abstract])) OR (((((((((((((co-design*[Title/Abstract]) OR (codesign*[Title/Abstract])) OR (participa* design*[Title/Abstract])) OR (co-creat*[Title/Abstract])) OR (cocreat*[Title/Abstract])) OR (co-produc*[Title/Abstract])) OR (coproduc*[Title/Abstract])) OR ((stakehold*[Title/Abstract]) AND (((engag*[Title/Abstract]) OR (involv*[Title/Abstract])) OR (collaborat[Title/Abstract])))) OR ((consumer*[Title/Abstract]) AND (((engag*'[Title/Abstract]) OR (involv*[Title/Abstract])) OR (collaborat*[Title/Abstract])))) OR ((participant[Title/Abstract]) AND (((engag*[Title/Abstract]) OR (involv*[Title/Abstract])) OR (collaborat*[Title/Abstract])))) OR (patient and public involv*[Title/Abstract])) OR (public contribut*[Title/Abstract])) AND (((((((("Research"[Mesh]) OR ("Clinical Trials as Topic"[Mesh])) OR ("Research Personnel"[Mesh])) OR (research[Title/Abstract])) OR (clinical trial*[Title/Abstract])) OR (clinical study[Title/Abstract])) OR (clinical studies[Title/Abstract])) OR (science[Title/Abstract]))) | 61,922 |
| **Attitudes**  ((((((((((((((("Attitude"[Mesh]) OR (Attitude[Title/Abstract])) OR (experience[Title/Abstract])) OR (experiences[Title/Abstract])) OR (perception[Title/Abstract])) OR (perceptions[Title/Abstract])) OR (perspective[Title/Abstract])) OR (perspectives[Title/Abstract])) OR (view[Title/Abstract])) OR (views[Title/Abstract])) OR (opinion[Title/Abstract])) OR (opinions[Title/Abstract])) OR (reflection[Title/Abstract])) OR (reflections[Title/Abstract])) OR (insight[Title/Abstract])) OR (insights[Title/Abstract]) | 3,966,364 |
| **Qualitative Research (Search filter from FMD(3S)/CHLA Canadian Health Libraries Association Federation des milieux documentaires section santé et service sociaux)**  (((“semi-structured”[TIAB] OR semistructured[TIAB] OR unstructured[TIAB] OR informal[TIAB] OR “in-depth”[TIAB] OR indepth[TIAB] OR “face-to-face”[TIAB] OR structured[TIAB] OR guide[TIAB] OR guides[TIAB]) AND (interview*[TIAB] OR discussion*[TIAB] OR questionnaire*[TIAB])) OR (“focus group”[TIAB] OR “focus groups”[TIAB] OR qualitative[TIAB] OR ethnograph*[TIAB] OR fieldwork[TIAB] OR “field work”[TIAB] OR “key informant”[TIAB])) OR “interviews as topic”[Mesh] OR “focus groups”[Mesh] OR narration[Mesh] OR qualitative research[Mesh] OR "personal narratives as topic"[Mesh] | 697,033 |
| **Caregivers AND Participatory research OR (co-design AND research) AND Attitudes AND Qualitative Research**  ((((((((((((((((("Caregivers"[Mesh]) OR ("Family"[Mesh])) OR (caregivers[Title/Abstract])) OR (family[Title/Abstract])) OR (families[Title/Abstract])) OR (mothers[Title/Abstract])) OR (mother[Title/Abstract])) OR (fathers[Title/Abstract])) OR (father[Title/Abstract])) OR (carers[Title/Abstract])) OR (carer[Title/Abstract]))) OR (relatives[Title/Abstract])) OR (relative[Title/Abstract])) OR (next of kin[Title/Abstract])) AND (Participatory research OR (co-design AND research) ((((((("Community-Based Participatory Research"[Mesh]) OR (Participatory Research[Title/Abstract])) OR (citizen science[Title/Abstract])) OR (co-researcher[Title/Abstract])) OR (coresearcher[Title/Abstract])) OR (co-researchers[Title/Abstract])) OR (coresearchers[Title/Abstract])) OR (((((((((((((co-design*[Title/Abstract]) OR (codesign*[Title/Abstract])) OR (participa* design*[Title/Abstract])) OR (co-creat*[Title/Abstract])) OR (cocreat*[Title/Abstract])) OR (co-produc*[Title/Abstract])) OR (coproduc*[Title/Abstract])) OR ((stakehold*[Title/Abstract]) AND (((engag*[Title/Abstract]) OR (involv*[Title/Abstract])) OR (collaborat[Title/Abstract])))) OR ((consumer*[Title/Abstract]) AND (((engag*'[Title/Abstract]) OR (involv*[Title/Abstract])) OR (collaborat*[Title/Abstract])))) OR ((participant[Title/Abstract]) AND (((engag*[Title/Abstract]) OR (involv*[Title/Abstract])) OR (collaborat*[Title/Abstract])))) OR (patient and public involv*[Title/Abstract])) OR (public contribut*[Title/Abstract])) AND (((((((("Research"[Mesh]) OR ("Clinical Trials as Topic"[Mesh])) OR ("Research Personnel"[Mesh])) OR (research[Title/Abstract])) OR (clinical trial*[Title/Abstract])) OR (clinical study[Title/Abstract])) OR (clinical studies[Title/Abstract])) OR (science[Title/Abstract]))))) AND (((((((((((((((("Attitude"[Mesh]) OR (Attitude[Title/Abstract])) OR (experience[Title/Abstract])) OR (experiences[Title/Abstract])) OR (perception[Title/Abstract])) OR (perceptions[Title/Abstract])) OR (perspective[Title/Abstract])) OR (perspectives[Title/Abstract])) OR (view[Title/Abstract])) OR (views[Title/Abstract])) OR (opinion[Title/Abstract])) OR (opinions[Title/Abstract])) OR (reflection[Title/Abstract])) OR (reflections[Title/Abstract])) OR (insight[Title/Abstract])) OR (insights[Title/Abstract]))) AND ((("semi-structured"[Title/Abstract] OR "semistructured"[Title/Abstract] OR "unstructured"[Title/Abstract] OR "informal"[Title/Abstract] OR "in-depth"[Title/Abstract] OR "indepth"[Title/Abstract] OR "face-to-face"[Title/Abstract] OR "structured"[Title/Abstract] OR "guide"[Title/Abstract] OR "guides"[Title/Abstract]) AND ("interview*"[Title/Abstract] OR "discussion*"[Title/Abstract] OR "questionnaire*"[Title/Abstract])) OR ("focus group"[Title/Abstract] OR "focus groups"[Title/Abstract] OR "qualitative"[Title/Abstract] OR "ethnograph*"[Title/Abstract] OR "fieldwork"[Title/Abstract] OR "field work"[Title/Abstract] OR "key informant"[Title/Abstract]) OR "interviews as topic"[MeSH Terms] OR "focus groups"[MeSH Terms] OR "narration"[MeSH Terms] OR "qualitative research"[MeSH Terms] OR "personal narratives as topic"[MeSH Terms]) | 5567 |

**[Mesh]**: Searches subject headings in the Mesh term field. Explodes the Mesh term to include narrower Mesh terms.

**[Title/Abstract]**: Searches title and abstract fields.

***** = Truncation, searches for word variations

**“”** = Quotation Marks; searches for an exact phrase.

## **Cinahl (EBSCO) date searched 251128**

| **Caregivers**  (XB (next of kin)) OR (XB (relatives)) OR (XB (carers)) OR (XB (fathers)) OR (XB (mothers)) OR (XB (family)) OR (XB (caregivers)) OR (MH "Family+") OR (MH "Caregivers") | 774 541 |
| --- | --- |
| **Participatory Research**  **(**MH "Citizen Science") OR (XB (Participatory Research)) OR (XB (Citizen Science)) OR (XB (Co-researcher)) OR (XB (Coresearchers)) | 6 897 |
| **Co-design**  (XB (co-design*)) OR (XB (codesign*)) OR (XB (participa* design*)) OR (XB (co-creat*)) OR (XB (cocreat*)) OR (XB (co-produc*)) OR (XB (coproduc*)) OR (XB (stakehold* (engag* OR involv* OR colaborat*))) OR (XB (consumer* (engag* OR involv* OR colaborat*))) OR (XB (participant (engag* OR involv* OR colaborat*))) OR (XB (patient and public involv*)) OR (XB (public contribut*)) | 75 488 |
| **Research**  **(**MH "Research+") OR (MH "Clinical Trials+") OR (MH "Research Personnel+") OR (XB (research)) OR (XB (clinical trial*)) OR (XB (clinical study)) OR (XB (science)) | 3 876 094 |
| **CO-design AND research**  ((XB (co-design*)) OR (XB (codesign*)) OR (XB (participa* design*)) OR (XB (co-creat*)) OR (XB (cocreat*)) OR (XB (co-produc*)) OR (XB (coproduc*)) OR (XB (stakehold* (engag* OR involv* OR colaborat*))) OR (XB (consumer* (engag* OR involv* OR colaborat*))) OR (XB (participant (engag* OR involv* OR colaborat*))) OR (XB (patient and public involv*)) OR (XB (public contribut*))) AND ((MH "Research+") OR (MH "Clinical Trials+") OR (MH "Research Personnel+") OR (XB (research)) OR (XB (clinical trial*)) OR (XB (clinical study)) OR (XB (science))) | 66 250 |
| **Participatory research OR (co-design AND research)**  ((MH "Citizen Science") OR (XB (Participatory Research)) OR (XB (Citizen Science)) OR (XB (Co-researcher)) OR (XB (Coresearchers))) OR (((XB (co-design*)) OR (XB (codesign*)) OR (XB (participa* design*)) OR (XB (co-creat*)) OR (XB (cocreat*)) OR (XB (co-produc*)) OR (XB (coproduc*)) OR (XB (stakehold* (engag* OR involv* OR colaborat*))) OR (XB (consumer* (engag* OR involv* OR colaborat*))) OR (XB (participant (engag* OR involv* OR colaborat*))) OR (XB (patient and public involv*)) OR (XB (public contribut*))) AND ((MH "Research+") OR (MH "Clinical Trials+") OR (MH "Research Personnel+") OR (XB (research)) OR (XB (clinical trial*)) OR (XB (clinical study)) OR (XB (science)))) | 71 578 |
| **Attitudes**  (MH "Attitude+") OR (XB (Attitude)) OR (XB (experience)) OR (XB (perception)) OR (XB (perspective)) OR (XB (view)) OR (XB (opinion)) OR (XB (reflection)) OR (XB (insight)) | 1 374 009 |
| **Qualitative Studies (Adapted from Pubmed using ChatGPT)**  (((XB "semi-structured" OR XB semistructured OR XB unstructured OR XB informal OR XB "in-depth" OR XB indepth OR XB "face-to-face" OR XB structured OR XB guide OR XB guides) AND (XB interview* OR XB discussion* OR XB questionnaire*)) OR (XB "focus group" OR XB "focus groups" OR XB qualitative OR XB ethnograph* OR XB fieldwork OR XB "field work" OR XB "key informant")) OR MH "Interviews+" OR MH "Focus Groups" OR MH "Narratives" OR MH "Qualitative Studies+") | 530 566 |
| **Caregivers AND Participatory research OR (co-design AND research) AND Attitudes AND Qualitative Studies**  ((XB (next of kin)) OR (XB (relatives)) OR (XB (carers)) OR (XB (fathers)) OR (XB (mothers)) OR (XB (family)) OR (XB (caregivers)) OR (MH "Family+") OR (MH "Caregivers")) AND (((MH "Citizen Science") OR (XB (Participatory Research)) OR (XB (Citizen Science)) OR (XB (Co-researcher)) OR (XB (Coresearchers))) OR (((XB (co-design*)) OR (XB (codesign*)) OR (XB (participa* design*)) OR (XB (co-creat*)) OR (XB (cocreat*)) OR (XB (co-produc*)) OR (XB (coproduc*)) OR (XB (stakehold* (engag* OR involv* OR colaborat*))) OR (XB (consumer* (engag* OR involv* OR colaborat*))) OR (XB (participant (engag* OR involv* OR colaborat*))) OR (XB (patient and public involv*)) OR (XB (public contribut*))) AND ((MH "Research+") OR (MH "Clinical Trials+") OR (MH "Research Personnel+") OR (XB (research)) OR (XB (clinical trial*)) OR (XB (clinical study)) OR (XB (science))))) AND ((MH "Attitude+") OR (XB (Attitude)) OR (XB (experience)) OR (XB (perception)) OR (XB (perspective)) OR (XB (view)) OR (XB (opinion)) OR (XB (reflection)) OR (XB (insight))) AND ((((XB "semi-structured" OR XB semistructured OR XB unstructured OR XB informal OR XB "in-depth" OR XB indepth OR XB "face-to-face" OR XB structured OR XB guide OR XB guides) AND (XB interview* OR XB discussion* OR XB questionnaire*)) OR (XB "focus group" OR XB "focus groups" OR XB qualitative OR XB ethnograph* OR XB fieldwork OR XB "field work" OR XB "key informant")) OR MH "Interviews+" OR MH "Focus Groups" OR MH "Narratives" OR MH "Qualitative Studies+")) | 5422 |

**MH** = Exact Subject Heading from CINAHL Subject Headings.

**MH+ =** Exact Subject Heading from CINAHL Subject Headings. Explodes the Subject Heading to include narrower terms.

**XB**: Searches title and abstract fields.

***** = Truncation, searches for word variations**.**

**“”** = Quotation Marks; searches for an exact phrase.

## **PsycInfo (EBSCO) date searched 251128**

| **Caregivers** |  |
| --- | --- |
| (DE "Caregivers+") OR (DE "Family+") OR (XB (caregivers)) OR (XB (caregiver)) OR (XB (family)) OR (XB (families)) OR (XB (mothers)) OR (XB (mother)) OR (XB (fathers)) OR (XB (father)) OR (XB (carers)) OR (XB (carer)) OR (XB (relatives)) OR (XB (relative)) OR (XB ("next of kin")) | 809 389 |
| **Participatory Research** |  |
| (DE "Community-Based Participatory Research") OR (XB ("Participatory Research")) OR (XB ("citizen science")) OR (XB ("co-researcher")) OR (XB (coresearcher)) OR (XB ("co-researchers")) OR (XB (coresearchers)) | 6 691 |
| **Co-design** |  |
| (XB (co-design*)) OR (XB (codesign*)) OR (XB (participa* design*)) OR (XB (co-creat*)) OR (XB (cocreat*)) OR (XB (co-produc*)) OR (XB (coproduc*)) OR (XB (stakehold* (engag* OR involv* OR colaborat*))) OR (XB (consumer* (engag* OR involv* OR colaborat*))) OR (XB (participant (engag* OR involv* OR colaborat*))) OR (XB (patient and public involv*)) OR (XB (public contribut*)) | 81 593 |
| **Research** |  |
| (DE "Experimentation+") OR (DE "Clinical Trials+") OR (XB (research)) OR (XB ("clinical trial*")) OR (XB ("clinical study") OR (XB science)) | 1 519 864 |
| **Co-design AND Research** |  |
| ((XB (co-design*)) OR (XB (codesign*)) OR (XB (participa* design*)) OR (XB (co-creat*)) OR (XB (cocreat*)) OR (XB (co-produc*)) OR (XB (coproduc*)) OR (XB (stakehold* (engag* OR involv* OR colaborat*))) OR (XB (consumer* (engag* OR involv* OR colaborat*))) OR (XB (participant (engag* OR involv* OR colaborat*))) OR (XB (patient and public involv*)) OR (XB (public contribut*))) AND ((DE "Experimentation+") OR (DE "Clinical Trials+") OR (XB (research)) OR (XB ("clinical trial*")) OR (XB ("clinical study") OR (XB science))) | 38 595 |
| **Participatory Research OR (Co-design AND Research)** |  |
| ((DE "Community-Based Participatory Research") OR (XB ("Participatory Research")) OR (XB ("citizen science")) OR (XB ("co-researcher")) OR (XB (coresearcher)) OR (XB ("co-researchers")) OR (XB (coresearchers))) OR (((XB (co-design*)) OR (XB (codesign*)) OR (XB (participa* design*)) OR (XB (co-creat*)) OR (XB (cocreat*)) OR (XB (co-produc*)) OR (XB (coproduc*)) OR (XB (stakehold* (engag* OR involv* OR colaborat*))) OR (XB (consumer* (engag* OR involv* OR colaborat*))) OR (XB (participant (engag* OR involv* OR colaborat*))) OR (XB (patient and public involv*)) OR (XB (public contribut*))) AND ((DE "Experimentation+") OR (DE "Clinical Trials+") OR (XB (research)) OR (XB ("clinical trial*")) OR (XB ("clinical study") OR (XB science)))) | 44 268 |
| **Attitudes** |  |
| (DE "Attitudes+" OR XB attitude OR XB experience OR XB experiences OR XB perception OR XB perceptions OR XB perspective OR XB perspectives OR XB view OR XB views OR XB opinion OR XB opinions OR XB reflection OR XB reflections OR XB insight OR XB insights) | 1 777 692 |
| **Qualitative Research** |  |
| (((XB "semi-structured" OR XB semistructured OR XB unstructured OR XB informal OR XB "in-depth" OR XB indepth OR XB "face-to-face" OR XB structured OR XB guide OR XB guides) AND (XB interview* OR XB discussion* OR XB questionnaire*)) OR (XB "focus group" OR XB "focus groups" OR XB qualitative OR XB ethnograph* OR XB fieldwork OR XB "field work" OR XB "key informant")) OR DE "Interviews+" OR DE "Narratives" OR DE "Qualitative Methods+") | 469 050 |
| **Caregivers AND** **Participatory Research OR (Co-design AND Research) AND Attitudes AND Qualitative Method**  ((DE "Caregivers+" OR DE "Family+" OR XB caregivers OR XB family OR XB families OR XB mothers OR XB mother OR XB fathers OR XB father OR XB carers OR XB carer OR XB relatives OR XB relative OR XB "next of kin")) AND (((DE "Community-Based Participatory Research" OR XB "Participatory Research" OR XB "citizen science" OR XB "co-researcher" OR XB coresearcher OR XB "co-researchers" OR XB coresearchers)) OR (((XB (co-design*)) OR (XB (codesign*)) OR (XB (participa* design*)) OR (XB (co-creat*)) OR (XB (cocreat*)) OR (XB (co-produc*)) OR (XB (coproduc*)) OR (XB (stakehold* (engag* OR involv* OR colaborat*))) OR (XB (consumer* (engag* OR involv* OR colaborat*))) OR (XB (participant (engag* OR involv* OR colaborat*))) OR (XB (patient and public involv*)) OR (XB (public contribut*))) AND ((DE "Experimentation+" OR DE "Clinical Trials+" OR XB research OR XB "clinical trial*" OR XB "clinical study" OR XB science)))) AND ((DE "Attitudes+" OR XB attitude OR XB experience OR XB experiences OR XB perception OR XB perceptions OR XB perspective OR XB perspectives OR XB view OR XB views OR XB opinion OR XB opinions OR XB reflection OR XB reflections OR XB insight OR XB insights)) AND ((((XB "semi-structured" OR XB semistructured OR XB unstructured OR XB informal OR XB "in-depth" OR XB indepth OR XB "face-to-face" OR XB structured OR XB guide OR XB guides) AND (XB interview* OR XB discussion* OR XB questionnaire*)) OR (XB "focus group" OR XB "focus groups" OR XB qualitative OR XB ethnograph* OR XB fieldwork OR XB "field work" OR XB "key informant")) OR DE "Interviews+" OR DE "Narratives" OR DE "Qualitative Methods+")) | 2260 |

**DE** = Exact Subject Heading from APA Thesaurus.

**DE+** = Exact Subject Heading from APA Thesaurus. Explodes the Subject Heading to include narrower terms.

**XB**: Searches title and abstract.

***** = Truncation, searches word variations

**“”** = Quotation Marks; searches for an exact phrase.
