# Supplementary material for: Family caregivers’ experiences of involvement in co-design research: A qualitative systematic review to develop involvement recommendations
Source: Int J Nurs Stud Adv. 2026 Nov 2;11:100730. doi: 10.1016/j.ijnsa.2026.100730 (PMC13770637; doi:10.1016/j.ijnsa.2026.100730)
Supplement: Supplementary file 2 [file mmc2.docx]

Supplemental file 3 | Critical appraisal of included studies

| **Qualitative design** | Design and method | | | | | | | Participants | Ethics | Results |
| --- | --- | --- | --- | --- | --- | --- | --- | --- | --- | --- |
|  | Q1 | Q2* | Q3* | Q4* | Q5 | Q6* | Q7* | Q8 | Q9 | Q10 |
| **First author et al. (year)** |  |  |  |  |  |  |  |  |  |  |
| Beighton et al. (2019) |  |  |  |  |  |  |  |  |  |  |
| Clarke et al. (2025) |  |  |  |  |  |  |  |  |  |  |
| Clarke et al. (2021) |  |  |  |  |  |  |  |  |  |  |
| Coupe & Mathieson (2020) |  |  |  |  |  |  |  |  |  |  |
| Di Lorito et al. (2020) |  |  |  |  |  |  |  |  |  |  |
| Eaton England et al. (2021) |  |  |  |  |  |  |  |  |  |  |
| Gutman et al. (2022) |  |  |  |  |  |  |  |  |  |  |
| Harrisson & Palmer (2015) |  |  |  |  |  |  |  |  |  |  |
| Hemphill et al. (2020) |  |  |  |  |  |  |  |  |  |  |
| Hudson et al. (2025) |  |  |  |  |  |  |  |  |  |  |
| Lord et al. (2022) |  |  |  |  |  |  |  |  |  |  |
| Lucchese et al. (2024) |  |  |  |  |  |  |  |  |  |  |
| Masoud et al. (2021) |  |  |  |  |  |  |  |  |  |  |
| Miah et al. (2020) |  |  |  |  |  |  |  |  |  |  |
| Musson et al. (2019) |  |  |  |  |  |  |  |  |  |  |
| Nygaard et al. (2020) |  |  |  |  |  |  |  |  |  |  |
| Sutherland et al. (2025) |  |  |  |  |  |  |  |  |  |  |
| Yamaguchi et al. (2022) |  |  |  |  |  |  |  |  |  |  |

*Note:* Color codes: green=”yes”, red=”no”, yellow=”unclear” *Items used of rating of dependability (Munn et al., 2014).
