# Supplementary material for: Family caregivers’ experiences of involvement in co-design research: A qualitative systematic review to develop involvement recommendations
Source: Int J Nurs Stud Adv. 2026 Nov 2;11:100730. doi: 10.1016/j.ijnsa.2026.100730 (PMC13770637; doi:10.1016/j.ijnsa.2026.100730)
Supplement: Supplementary file 3 [file mmc3.docx]

**Supplemental file 4.** Findings and supporting data extracts.

| **Findings** | **Level of support** | **Data extracts** |
| --- | --- | --- |
| Improving people's lives as a motivator | U | Partners reported wanting to help improve people's lives and patient outcomes …  “When my mom had her stroke I was the decision maker. I will never forget how stressful that was. I want to help make the entire process as easy as it can possibly be for both the family member and the patient” |
| Improving health-care experiences as a motivator | C | Similarly, partners wanted to improve healthcare experiences and patient‐provider communication |
| Addressing a gap for underserved communities as a motivator | U | Partners noted that the population or condition of focus in the research project was understudied, underserved, poorly understood or had an unmet need. Examples of underserved populations included  Latina, Black male, elderly, rural and native populations, as well as those with less access to specific types of health care. ”This project will fill a need of families in remote parts of the country, and someday the world, by connecting them with current up‐to‐date information that will help them support their children’. |
| Believing in the value of the health intervention as a motivator | C | Partners expressed a belief that the studied health intervention or treatment would help patients and families, and some were motivated by prior positive experience with the intervention. |
| Representing a perspective and having a voice in research as a motivator | C | Partners indicated the importance of people with their backgrounds or experiences participating in research that affects their lives.  One patient partner stated, “patients have a unique perspective on healthcare and can provide important information for improving  Healthcare”. Partners also characterized this as ‘having a voice’ in research. |
| All perspectives should be represented | C | Some expressed that all perspectives, such as patient, caregiver and clinician, should be represented in research. |
| Research partners at the table with researchers as co‐creators as important | C | For instance, one partner representing a community‐based organization said, ‘This project places research partners at the table with researchers as co‐creators of knowledge and research |
| Wants to learn more about the condition and care as a motivator | C | Partners expressed personal, professional or scientific curiosity and desire to learn about the study topic. Patient partners and caregivers typically wanted to learn more about their condition  or about research that addressed issues relevant to their care. |
| Participation means a pause from caregiving | C | Additionally, one caregiver partner mentioned more practical reasons for contributing: ‘If I could help any. It gave me an hour or so away from  my wife, who has dementia and Parkinson's. Plus, they paid me $150’. |
| Financial compensation as an incentive | C | Additionally, one caregiver partner mentioned more practical reasons for contributing: “If I could help any. It gave me an hour or so away from  my wife, who has dementia and Parkinson's. Plus, they paid me $150”’ |
| Making a difference makes | U | It is also a good feeling to know that our Board has helped to improve the treatment of other geriatric cancer patients” |
| Building new or better relationships as a benefit | C | Partners indicated developing new relationships as a result of engaging in research. For example, partners described connecting with and learning from other patients and caregivers and drawing  strength from that experience ”The bond of understanding I have with the other involved parents is reassuring. Knowing that we all have the same struggles and knowing that we are helping to make some of these struggles easier for other families is very rewarding’ |
| Developing greater knowledge and appreciation of research as a benefit | C | Partners discussed gaining an improved understanding of research  and wanting to continue being involved in research |
| Positive experience being part of the research team and being listened to | U | Partners also reported having a positive experience as part of the research team “This is the first opportunity that I have had the privilege to be a part of where the researchers really wanted to know what families think and to listen to their real‐life stories” |
| Participation led to self-improvement | US | Partners described several ways their experience led to self‐improvement, such as gaining confidence or becoming a better teammate. |
| Participation did not lead to any changes in their lives | US | Some partners reported that engaging in research had not led to any changes in their lives. While most of these partners did not expand on their answer, some noted they still had a positive experience as a research partner |
| Supportive relationships were developed with other participants | U | Supportive relationships were developed with other lay members, group leaders and other researchers and clinicians. Shared experiences of stroke were perceived to strengthen the relationships  between lay members. “Yeah, the social side is very good. Everybody is very friendly and everybody has a common cause and I think that binds people together” |
| The relationships participants developed with other researchers and clinicians were held in high regard | C | Participants spoke with equal warmth about the professionals that lead the different advisory groups, describing them as ‘‘very good acquaintances’’ or ‘‘friends’’. The relationships participants developed with other researchers and clinicians were held in high regard because they allowed them to associate with an intellectual  and professional elite. |
| The benefits of intellectual stimulation | US | The benefits of intellectual stimulation were divided into those who wanted to keep learning for self-empowerment, as one participant said ‘‘knowledge is power’’, and those who wanted to continue challenging themselves intellectually to aid their recovery from their stroke. |
| Bringing a different perspective | C | The most frequently described beneficial impact of PPI to the research process was bringing a different perspective. |
| A combination of experiential and professional expertise was synergetic | C | Participants thought the synergy of the experiential expertise of stroke survivors with the professional expertise of researchers and clinicians benefitted the research process. |
| Challenging the researcher perspective  22 | U | Participants described asking questions to challenge the researcher’s assumptions; some participants described this resulting in changes to the design of the research or the abandonment of  a proposal.  ”Well sometimes people can have an idea for a research and it sounds very good on paper and then they’ll bring it to a group like ours and we’ll say ‘well, what really are you intending?’ and almost take the wind out of their sails sometimes!  [laughing] And we will say ‘well how will it benefit, what will it do?’ and when it comes down to it, it doesn’t really, it just sounded a good idea” |
| Credibility arose from questions about training, the lay member role and representativeness and was based on the trust and value placed on the participants’ contributions | C | The notion of credibility arose from questions about training, the lay member role and representativeness and was based on the trust  and value placed on the participants’ contributions by professionals and other stroke survivors. |
| Representativeness was requested | C | Representativeness was thought to be increased through the incorporation of a wide range of diverse perspectives within an advisory group. |
| Increased involvement would feel like a job and would become stressful | C | Other participants felt that their level of involvement was appropriate at that time and stated barriers to becoming more  involved, including: time demand, lack of computer skills and internet and age. Furthermore one participant suggested that greater involvement would make it feel like a job.  “I’ve enjoyed doing what I’ve done but if it was anything more it would become a job and it would become stressful” |
| Provides respite for carers as a benefit | C | ”But also it’s the just getting away from it, you know and having a piece of cake and a cup of tea!” |
| Training in research skills would detract from why they were in the group because they would no longer be lay member | C | Those participants that placed greatest value on their experiential knowledge did not want training or even felt that training might detract from their lay role. “If we had training in research skills I think that would detract from why we’re in the group because we would no longer be lay members” |
| Supportive relationships as a facilitator | C | The most frequently discussed facilitator was the importance of supportive relationships with group facilitators and other group members. Supportiveness of group facilitators was attributed to appropriate training, whereas the support from the group was described in terms of friendship and togetherness. |
| Giving something back | US | Patientcentered |
| Doing something useful | US | Patientcentered |
| Gaining confidence | C | Good for the patient |
| Intellectual stimulation | US | Patientcentered |
| Negative impact of PPI | US | Patientcentered |
| Bringing a different perspective | US | Patientcentered |
| Patient fatigue as a barrier | C | Not tiring for me, it’s tiring for such as Berol or [name of other stroke survivor], they get tired after  2 hours. |
| Holding meetings in disabled-friendly environments to facilitate | C | The following factors facilitated participation for  individual respondents: holding meetings in disabled-friendly environments, |
| Transportation aid as facilitating | C | Practical facilitators such as providing transport and  meeting in accessible environments were viewed as essential by those patients with mobility difficulties and those who are unable to drive after their stroke.  “And I think one lady is provided with a taxi to get there because she can’t drive”. |
| Participants were sceptical about whether researchers truly valued their involvement | C | Researchers treated lay members with respect and many members felt that they had equal relationships with the professionals.  However, some participants were sceptical about whether researchers truly valued their involvement. Most participants expressing this view worked at all three levels of involvement, potentially suggesting that professional’s scepticism increases when lay members play a role in controlling the research. |
| Barriers to involvement: time demand | U | Patients wanted to be more involved or was fine with it as was!  I have enjoyed doing what I have done, but if it was anything more it would become a job and it would become stressful |
| Participation as respite | C | But also it’s the just getting away from it, you know and having a piece of cake and a cup of tea!  (Joyce) |
| Not being able to leave the patient unattended is a barrier | C | Furthermore, the following barriers were raised by individual respondents: physical limitation of dealing with paperwork with one hand, carers unable to leave the patient unattended and the  consequences of age coupled with stroke |
| Giving a voice to those who are often unheard | U | Family carers expressed their motivation to support research and enhance the knowledge and understanding of adults with a mild intellectual disability who display aggressive challenging behaviour. Family carers empha­sised the importance of the voices of adults with a mild intellectual disability being heard and how this should influence the direction of research, particularly for those with social and communication needs whose insight is often not well represented.  “I have a brother who uses aggressive challenging behaviour. He has a severe learning disability so get­ting his views directly would be difficult so I think there’s definitely an advocacy role for me in terms of sharing some of that experience of his.”  “Sometimes in a world like we live in today, children like [mine’s] voices are lost so I can be that voice.” (Family carer 2 |
| Altruistic desire to help | U | Similarly, family carers described their participation in the PETAL PPIE activities as being driven by a desire to support other families to improve their quality of life and subsequently the well-being of adults with an intellectual disability who display aggressive challenging behaviour. The financial incentives were appreciated but played a minimal role in their decision to participate in research.  “I work very hard to try and improve the system for people like my son to make sure that their needs are better understood.” (Family carer 3).  “I’ve been offered… remuneration a lot of times and that has been…well received… I don’t actually take the payments because I do it voluntarily…I get such a lot of satisfaction out of helping with projects you know like this that’s payment enough for me.” (Fam­ily carer 3). |
| Knowledge exchange in caregiving as beneficial  44 | U | The exchange of knowledge as part of the research pro­gramme was helpful not only from the viewpoint of the researchers but also by the members of the PPIE groups, specifically around ways in which they could better sup­port those they care for.  “I learned other things from the other contributors as well because when I watched the videos of them doing their talk or explaining how they supported their family member I thought oh… that’s a great idea I must try that.” (Family carer 2). |
| Guidance and consensus building relating to the use of terms and language used within meetings and study documentation as a significant contribution | U | One of the most important ongoing contributions of the PPIE groups has been the guidance and consensus building relating to the use of terms and language used within meetings and study documentation.  “The language being used was I felt inappropriate in some cases the use of the word aggressive and treat­ment were two of the things that kind of touched a nerve because…if you ask somebody…what’s aggres­sive they see it as a very negative thing most people will if you don’t give them the context and even if you give them the context sometimes they won’t understand unless they’ve had the experience them­selves.” |
| Offering different meeting dates and times and taking into consideration the caring responsibilities of family carers was appreciated | U | Family carers expressed how researchers were supportive and open to making attendance as convenient as possible, offering different meeting dates and times and taking into consideration the caring responsibilities of family carers.  “Normally…there are…2 or 3 suggestions about tim­ings for meetings and plenty of notice has been given as it’s online… I have been away once but I could still join the meeting online so it wasn’t a problem. You’re never gonna get a suitable time for everybody. But certainly our opinion is asked for about when meetings take place it can be flexible as possible.” |
| Further discussions on practicalities prior to involvement were requested. | C | However, one family carer raised that there was limited discussion prior to their involvement about what would work best for the entire PPIE group. “There wasn’t enough discussion… probably about… maybe halving the group and having some in the daytime and some on an evening.” |
| The format of meetings may not have been entirely what some family carers originally anticipated when they joined the study | U | The format of meetings may not have been entirely what some family carers originally anticipated when they joined the study. However, this change in expectations was beyond the research team’s control due to the start of the COVID-19 pandemic in March 2020. “I mean it was just described as giving a lived expe­rience as a family carer of somebody who has aggres­sive challenging behaviour…so it was pre-pandemic when it was set up so we didn’t know that all the meetings were going to be online at the beginning… so yeah from that point of view it’s not been quite as I expected.” |
| Not aware of the extent of the commitment that was necessary to be involved | U | one family carer was not aware of the extent of the commitment that was necessary to be involved in this research. This meant that they were not always able to dedicate the full amount of time required to take part in meetings.  “I don’t think I had an understanding beforehand of how much of a commitment it was gonna be how frequent the meetings would be which had I had that I think I would have known that I couldn’t give the time required.” |
| Range of perspectives from different caregiver roles in important | U | Family carers reported there was a good range of per­spectives within the group in terms of caregiving roles. Family carers stated that they were provided with oppor­tunities and encouragement to share their opinions and perspectives.  “I think overall as a sibling I have a slightly different perspective… so for me it’s about making sure that there’s that sort of perspective that understands the needs of siblings as a different need and as a sepa­rate need but also the understanding and insight of siblings as slightly different and slightly separate to those of parents because it is a different relation­ship.” |
| Their views were genuinely valued by the research team | U | Adults with a mild intellectual disability and family carers indicated their views were genuinely valued by the research team throughout their involvement.  “I thought that not only our voices were heard but they were listened to… the team made sure that they knew why we were making those comments-why I was making those comments because that’s so important you know understanding the why’s and the wherefores and so on and lived experience actu­ally gives you the means with which to do that if you can only have time to be heard. And I felt that we were given time to be heard, and space to be heard so it was quite a positive experience.” ( |
| Experiences being incorporated into the study were positive. | U | The family carers group expressed their satisfaction once they realised their comments and ideas were being incorporated into the study. |
| FCs referred to the power of accessible, sim­ple and inclusive presentation of practices by researchers which made them feel more confident in their abili­ties to make meaningful contributions. | U | Family car­ers, in particular, referred to the power of accessible, sim­ple and inclusive presentation of practices by researchers. This made family carers feel more confident in their abili­ties to make meaningful contributions that would have an impact on the research. “Not all of us feel confident that we can come and do this but I think if something is put in that format and it’s not frightening and it doesn’t look like well could I do it? They think well you know what I could take part in that study cause I would like my daugh­ter to achieve that or my son to achieve that.” |
| FCs perceived their participation to be authentic and not tokenistic, with their contribution respected and seen as valid and important which was positive. | U | Both groups perceived their participation to be authentic and not tokenistic, with their contribution respected and seen as valid and important by the research team. They described feeling listened to and that there was a genuine interest in their experiences, leaving them feeling confident that their views would be carried forward. “I genuinely felt, and I’ve said this to various people, but this wasn’t just a tick box exercise, ooh yes, I’ve consulted carers, it was a genuine . . . let’s see how you can get involved and I’d like to incorporate your ideas in it, so it did feel like genuine involvement which was great |
| Aspiered to a collaborative/participatory rather than a consultative model of engagement. | C | This illustrates that our parent carers clearly aspired to a collaborative/participatory rather than a consultative model of engagement. They described how when suggestions were made by them, none were ever ‘shrugged off’ or dismissed as irrelevant |
| One of the main things I got from this was that he was interested in the project from our point of view, | U | “... one of the main things I got from this was that he was interested in the project from our point of view, what was useful, helpful and interesting to us as carers, not just the scientific community, and actually trying to get useful information from us for the studies, not just interesting scientific information. . . you know, looking at the mortality rates, looking at how GPs interact and check-ups and all of those/that’s the things I need to know about as her carer. |
| The aim and the methods of the research study and the ways in which they could provide input into the study were set out very clearly at the beginning | U | With the parent carers, this positive experience had stemmed from when they were initially consulted to see if they would be interested in participating. They recalled the aim and the methods of the research study and the ways in which they could provide input into the study were set out very clearly to them by the principal investigator  at the beginning |
| FCs felt that their suggestions and experience had made a strong contribution to the study, recounting how important it was to them to have been able to address issues of relevance to them and not just contribute to what the researchers wanted to study | U | The FCs felt that their suggestions and experience had made a strong contribution to the study, recounting how important it was to them to have been able to address issues of relevance to them and not just contribute to what the researchers wanted to study. “... one of the main things I got from this was that he was interested in the project from our point of view, what was useful, helpful and interesting to us as carers, not just the scientific community, and  actually trying to get useful information from us for the studies, not just interesting scientific information. . . you know, looking at the mortality rates, looking at how GPs interact and check-ups and all of those/that’s the things I need to know about as her carer |
| Contribution rewarding - Limitations frustrating | U | Contribution rewarding – Limitations (gp) frustrating |
| FCs expressed frustration that some of their suggestions could not be explored further due to the limitations of the information held on the GP database | U | Although the parent carers were pleased that their suggestions were taken on board, they also expressed frustration that some of their suggestions could not be explored further due to the limitations of the information held on the GP database, however this also contributed to them gaining a greater insight into the research process: “. . . the main thing we would have liked to have seen was more analysis by where people lived and who they lived with, whether they lived with their family, whether they lived independently, whether they lived in a residential care home, supported living, because we felt that that might explain quite a lot of the variations in things like umm how many people had health checks, how many people were taken to A&E etc . . . . “ There’s frustration of the data set and being able to pull out the kind of information we want answers to, but that wasn’t something that was wrong with the study |
| Recognition of the diversity of caregiving roles – yet shared experiences | U | The participants focused on their diversity, recognizing that carers differ as much as any other societal group in terms of age, resources, circumstances and family situation. Despite such differences, they also reported sharing many experiences. One participant said this: “Carers of persons with dementia are not a uniform group, so you’ll probably find as many different opinions there as you would in society in general.” |
| It was important to participate in the PSP process to share experiences with others in the same situation | U | Furthermore, the participants clearly emphasized that it was important to participate in the PSP process to share experiences with others in the same situation. They saw participation as a way of helping to relieve bad feelings, such as shame, for other carers. One participant said this: “Very many people I talk to say, they have felt that pain. I recognize it. I’m like you – I know something about being on the verge of shame. I’ve tasted the feeling of life being meaningless. Not to mention empty.’ I’m afraid of being exposed as a useless carer – I’ve felt like a total failure. Although I now feel confident. To share one’s innermost thoughts with others, your most shameful thoughts – I think that is of value to others.” |
| It was important to participate in the PSP process to share experiences with others in the same situation | U | The participants also said that it was common to experience joyful moments and share pleasant experiences with the person affected with dementia: “I want to say that all those years with Alzheimer’s, it was not only sorrow. On the contrary, there was a lot of happiness, many bright moments and much good. And I had my husband home until the end. I was younger, so I was able to.” |
| Given voice to the unheard is not a given | U | Facilitating participation in the PSP process by those with dementia and including their experiences may help to uncover research areas that have received inadequate attention. One participant pointed out the importance of sufferers’ voices being heard in research: “Previously we, as the carers, have been their spokespeople, right, but it’s not a given. If they’ve lost the ability to speak in an early phase, it’s not certain that we assume correctly, and if they don’t lose the ability to speak, it’s not really certain that we think the same way they’re thinking. So, if they’re allowed to express themselves without interference, it’s definitely much better, as long as they can.” |
| The carers and those with dementia should receive the information about the project needed for participation in the PSP process | U | In order for persons with dementia and their carers to be able to participate in the PSP process, support  must be provided if necessary for the carers. The participants were concerned that the carers and those with dementia should receive the information about the project needed for participation in the PSP process. They said that their involvement in the PSP process would be demanding in terms of the time and energy needed: “Of course, it can be taxing to participate in the whole process, which seems fairly long, and it isn’t easy to commit to participating in a process like this, that might last more than half a year, especially not in the period when you are most burdened.” |
| Need for facilitation – cannot leave the home without the person with dementia | U | Noting that carers needed energy to participate in a long PSP process, they said that facilitation had to be optimal for those with dementia and their carers. Some carers cannot leave the house without the person with dementia: “And you must facilitate participation. That was a big problem, I could barely leave my husband for long enough to get to the mailbox.” |
| Facilitation includes helping the participants to understand the project and clarifying the researchers’ expectations of the partnership | U | Facilitation includes helping the participants to understand the project and clarifying the researchers’ expectations of the partnership with the participants. One participant commented thus: “[It is necessary] to set aside time and resources for collaboration in the planning phase, and to clarify expectations as to how the service user should contribute. I imagine it could become difficult when it comes to interpreting the information gathered.” |
| Continuously participation in all PSP steps was highlighted by several participants, who emphasized that it could be difficult for people with dementia to participate in the entire PSP process | U | Continuously participation in all PSP steps was highlighted by several participants, who emphasized  that it could be difficult for people with dementia to participate in the entire PSP process. One participant said this:  “But carers should, if possible, contribute at all stages of the research process if they are willing. Naturally, challenges must be discussed openly all along. The person with dementia might not have the necessary resources to do it. I would think that the carers represent continuity [to the people with dementia]. They have the experience alongside the service users.” |
| The participants also emphasized the importance of carers being with the person with dementia throughout the PSP process because the disease would progress during the process | U | The participants also emphasized the importance of carers being with the person with dementia throughout the PSP process because the disease would progress during the process. One participant said the following: “I think you’ll get more service users in the early stages of the disease, before it’s very far gone, but that makes it important to include the carers all along, because if you are to follow a research process through all the stages, it will take time and the disease progresses all the time. The carer then provides the continuity [to the people with dementia], having the experiences alongside the service user. So, I think this – but I also think there will be some barriers when it comes to recruitment – because they have to have acknowledged at an early stage the fact that they have dementia.” |
| Being a carer and peer supporter means that they experience several areas about which they need more knowledge. They also want research to contribute to societal understanding of what it means to be affected by dementia. | U | The participants explained that understanding what it is like to live with dementia makes it easier to master their carer role. They reported that being a carer and peer supporter means that they experience several areas about which they need more knowledge. They also want research to contribute to societal understanding of what it means to be affected by dementia. “And understanding. Yes, research into how to create understanding. That others understand those who are affected with dementia – not just carers. Little understanding around. Family, friends, workplace, everywhere. Researching how understanding can be achieved would be interesting and important. I think that a society with appreciation for weakness would be a better society for most.” |
| Participants should be recruited through organizations, so that potential participants understand that this is a serious process | U | The focus group participants also said that PSP participants should be recruited through organizations, so that potential participants understand that this is a serious process. One participant said this: “I think it might be advantageous to recruit service users through an organization if you want the service user to be able to convey the needs of the group represented by the organization.” |
| Important being heard! Not just an alibi to gain funding | U | Important being heard! Not just an alibi to gain funding |
| The participants said that they wanted research to focus on optimizing everyday life for both the people with dementia and their carers. They also wanted improved knowledge for the most vulnerable carers | U | The participants said that they wanted research to focus on optimizing everyday life for both the people with dementia and their carers. They also wanted improved knowledge for the most vulnerable carers, children and young people, about how it is to care for a parent with dementia: “We talked about being a child, a youth with parents with dementia and I said that – Has any research been done into how they fare later in life, when they’ve grown up with, perhaps over a long period, a mother or a father with dementia? And I’m personally very concerned about this, put it that way, but I feel it would be interesting on a more general basis too.” |
| The researcher needs to speak using language that the participants can understand and must feel that the participants’ roles in the PSP process are important to address a disparity of power | C | The participants observed a disparity in power of knowledge and language across the different positions of researcher, health professional and patient. The researcher needs to speak using language that the participants can understand and must feel that the participants’ roles in the PSP process are important |
| It is important for the participants not to feel that they are token participants in the process | C | The participants noted that researchers often approach clinical practice with ready-made solutions and definitive answers, which may lead to tension between differing intentions. It is important for the participants not to feel that they are token participants in the process, an alibi for researchers to obtain funding, but rather that their experiences are important and that their involvement has an impact |
| Both carers and the people with dementia must be taken seriously and must experience a sense of having been heard | U | Participants in the four focus groups highlighted the need for awareness that tension between participants’ different purposes could influence carers’ motivations for engagement in the PSP process. Both carers and the people with dementia must be taken seriously and must experience a sense of having been heard. One participant commented thus: “If one contributes, one must be taken seriously. I gather that we are very popular with researchers because they will not receive funding if they don’t include service users. It’s rather scary. One doesn’t really know if research becomes more applicable with user involvement.” The participants explained that carers have years of experience,  which makes them the “experts” on their situation, as one participant said: “It’s what I know, and I feel that I have something to contribute for others in the same situation that I’ve been in for 25 years. I perceive that my experience is important. I also feel that others’ experiences are beneficial to me.” |
| To involve FCs, researchers must understand these user groups and  how to interact with them | U | To involve people with dementia and their carers, researchers must understand these user groups and how to interact with them. One participant commented thus: “We also know that researchers must behave in a down-to-earth manner, to gain people’s trust.” |
| These informants do not want simply to be the researcher’s funding alibi. They need to feel equal | C | These informants do not want simply to be the researcher’s funding alibi. They need to feel equal to the researcher, to be an important part of the research: “It should not just be an alibi in order to be granted research funding.” |
| They want to help make improvements for others in the future who are in the same situation | U | They know that today’s research may not benefit them or their situation, but they want to help make improvements for others in the future who are in the same situation: “So, our participation in this, it’s to be able to help  those coming after us. That’s the way it’ll be, so it becomes interesting to see if what we’ve contributed to the project can help others. That’s the way it has to be. Because we are past that stage, we could have just turned our backs and not cared, because we are done with all of this, but that’s not who we are. I want to contribute. I want to make a difference to those coming after us.” |
| FCs needed to understand their role and purpose in the process. For them, before joining a steering group, it was important that they understood what was expected of them | U | From the participants’ perspective, because the PSP process is demanding, they needed to understand their role and purpose in the process. For them, before joining a steering group, it was important that they understood what was expected of them in the PSP process: “Clear outlines. First of all, be clear on the purpose of user involvement in the project in question. What is the reason, what is the goal, in what phases is it most important and to what extent can they be involved? Project management must be clear about the service users’ role and clarify expectations as to the use of time, type of contribution, co-authoring, remuneration, etc.” |
| Involvement must have an impact | U | The participants wanted to have a real impact on research through genuine user involvement. Gaining knowledge and information about the PSP process was at the core of the participants’ feedback: “There must be a purpose to our participation, let’s put it that way. We must have a say in prioritizing.” |
| There must be something in it for the FCs too | U | The informants were divided as to whether it was enough to know that research might benefit people in a similar situation in the future or if it was necessary to experience benefits in the present. As one participant said it:  “There must be something in it for me as a service user too, sort of. Is this useful to me and my problems, to get answers?” |
| A follow-up was requested to know whether their contribution was used | U | Information and follow-up at the end of the project are also important to the participants. As one participant said, they wanted to know whether their contribution was used and what came out of the project: “Often you hear nothing afterwards. Complete silence. The information you’ve given is just stored in a database or something. Involvement and follow-up are very important. One must know the what, how and why.” |
| FCs wanted those in policy and health services to hear their voices. They noted that research must have consequences for both people with dementia and their carers. | U | Through their contribution to the PSP process, the participants wanted those in policy and health services to hear their voices. They noted that research must have consequences for both people with dementia and their carers; these consequences include policy changes in the health services: “The research must have some consequences, be able to lead to something within health and social care or in politics, not least for the people in question. Research on causality might be too optimistic. As previously mentioned, it is my opinion that grasping the experiences of those with dementia and their carers is important for facilitation and obtaining insight.” |
| The predominant motivation was a desire to help other | C | The predominant motivation was a desire to help others, even if it would not help them directly. “If you could help in any possible way to MND research or helping people. It's this that I wanted to do.” |
| Bring a personal perspective to research as a contribution | C | Others wanted to raise awareness of research/MND and bring their personal perspective to research. Some felt strongly that pwMND should have a voice in research. |
| Participants felt valued and the group had a positive impact on participants’ self-worth | C | Participants felt valued and the group had a positive impact on participants’ self-worth. “I benefit from it from feeling that I've actually done something to actually contribute.” |
| Interested in research | US |  |
| Learn about research | US |  |
| Give something back | US |  |
| Raise awareness of research in MND | US |  |
| Gain information | US |  |
| Help improve care and treatment | US |  |
| FCs enjoyed being part of a supportive group with shared goals | C | Members enjoyed being part of a supportive group with shared goals. “We had that shared experience and there was a lot of comradery.” |
| Participation tackled the sense of isolation caused by the illness | C | Face-to-face meetings gave members the opportunity to hear from others in the same position and tackled the sense of isolation caused by MND. |
| Lack of awareness and understanding about PPI as a main barrier to participation | C | The main barrier was a lack of awareness and understanding about PPI as people may be unsure of the contribution they could make. “Not knowing exactly what it involves… and feeling that they have nothing to offer.” |
| Some felt like outsiders in their group and anticipated that people might lack the confidence to join an already established group | C | Despite assimilating into the group, some felt somewhat of an outsider compared to founding members. Therefore, participants anticipated that people might lack the confidence to join an already established group |
| People might be discouraged from participating if they believed research to be impenetrable. | C | Participants recognised that people might be discouraged from participating if they believed research to be impenetrable |
| Informal induction could increase confidence | C | Participants agreed that an informal induction was useful for increasing people’s confidence |
| Face-to-face meetings, and presentations facilitated participation | C | Face-to-face meetings and presentations facilitated participation. |
| Some liked that the meetings took place at a research unit but it was intimidating for others | C | Some liked that the meetings took place at a research unit but one pwMND thought this might make people feel “intimidated.” |
| Time commitment and technology were seen as potential barriers to participation | C | Time commitments and difficulty using technology were seen as potential barriers |
| FCs felt they benefited so time commitments was not a barrier | C | Participants felt they benefitted so the time commitment was not a barrier to them |
| Participation can be emotionally challenging | US |  |
| The group as a “safe place” was important to give honest opinions | C | There was a strong sense of ownership and members felt bonded through their shared experiences and goals. Members agreed that the group was friendly and supportive, making it a “safe place” to give honest opinions. |
| Those who were not confident talking  about their feelings would find it difficult | C | Those who were not confident talking about their feelings would find it difficult. |
| Some mentioned that members need to be open to listening | C | Some mentioned that members need to be open to listening. |
| Training was seen as helpful for increasing members’ confidence and gaining an understanding of research | C | The training was seen as helpful for increasing members’ confidence and gaining an understanding of research. However, participants felt only basic training was needed. |
| Experiential expertise as valid contribution | C | that anyone with experience of MND could make a valid contribution. “The most important thing is knowing about MND!” |
| Developing a collaborative member–researcher partnership was key to effective PPI | C | Developing a collaborative member–researcher partnership was key to effective PPI |
| *Scientists using appropriate language and being open to questioning helped establish a relationship* | C | Participants thought scientists used appropriate language and were open to questioning, allowing them to establish a relationship. Conversely, one pwMND felt nervous about participating in the group after listening to a presentation where the researcher used complex jargon |
| Wanted to know what was expected | C | Members felt supported to gain the knowledge they needed to be effective partners. However, when asked to comment on research, members wanted to be told what was expected of them |
| Feedback was important to maintain partnership and made them feel worthwhile | C | Feedback from researchers was seen as important to maintain the partnership. Not all members recognised they received feedback (pwMND) but those who did, valued it because it made them feel worthwhile |
| Despite differences, having the same aim made the group work well | C | Members said the administrator helped the group run “smoothly” (MND Association volunteer) whilst the chairperson ensured the group kept to the agenda and avoided members dominating the discussion. Participants  thought the discussions enabled a good balance between individuals’ own experiences and those of others. Despite disagreements, members believed the group worked well because they had “the same aim” |
| Collaboration made FCs feel motivated | U | Working together with my PPI colleague and an academic who is motivating and committed to the ethos of lay research was fulfilling and gave us a feeling of worth and motivation. |
| Being involved, contributing knowledge gained from lived experiences, was fulfilling and made FCs feel had given back to the community | U | Being involved with the study through the whole process, contributing knowledge gained from lived experiences, was fulfilling and made me feel I had, in a small way, given back to the community. |
| Training sessions were very helpful in building confidence | U | The training sessions were very helpful. At the briefing meeting, attended by the academic researcher, my fellow layresearcher and myself, we discussed the structure of the interviews, and decided who would ask which questions and what was expected of us. […] When I left this meeting I felt far more confident that I could be of value to the research project. |
| Developing understandable questions | U | As lay researchers, we were able to give input into how the questions should be delivered, to ensure these would be understood and acceptable to both participants and carers. |
| Experiential expertise could clash with the academic´s views and needs | U | The experience my PPI colleague and I brought from careers outside the confines of academia sometimes clashed with the academics’ views and needs. For example, in relation to participant-fronting documents, I found it was difficult to balance academic language requirements while also ensuring understandable wording for participants. |
| Research team meetings could be daunting | U | Having the confidence in academic meetings to use my own lived experiences to support or challenge research also presented with challenges. Sitting with academics at research team meetings was at times daunting. |
| Academics using language unfamiliar to lay people made FCs feel inhibited to speak out | U | When the academics used language unfamiliar to lay people, I felt inhibited to speak out. This might have limited the added value of hearing my views as a lay person. |
| It is important that the academic researcher is open to using lay people and is not dismissive of their views | U | The main lessons I have learned from the experience is how important it is in people-centred research that the academic researcher is open to using lay people and is not dismissive of their views. |
| Personal thoughts had a positive effect and empowered the carer to speak about worries that may otherwise have been withheld | U | I was concerned that I had talked about my own experiences too much and had deflected from the purpose of the interviews. However, on reading the transcripts, it was apparent that my personal thoughts had a positive effect and empowered the carer to speak about worries that may otherwise have been withheld |
| Managing sensitive situations | U | In the context of data collection, meeting participants and carers in their homes to discuss personal information required a great deal of experience in managing sensitive situations |
| Protocols and models are needed | C | Co-research is still in its infancy stage, protocols and models need to be created to ensure good practice |
| When familiar with study aims and procedure, one can participate with confidence | U | We had been an integral part of development of PrAISED and became very familiar with its aims and procedures. Together with helping write the participant-facing material, my husband and I had modelled as participant and carer for the publicity material and exercises. All this meant that I could approach the interviews confident in my understanding of the study and committed to its success |
| Researchers embracing their roles and building confidence help them meet challenges positively | U | When we met with the academic researcher, who embraced our roles and ensured we were fully confident to make this further contribution, we knew we could positively meet the challenge |
| The opportunity to debrief with the academic researcher gave me further confidence in my skills and contributed to my experience of PPI working at its very best | U | The opportunity to debrief with the academic researcher, who brought his professional capacity as a psychologist into every discussion, was a new experience for me and gave me further confidence in my skills. This is rarely if ever given to PPI, but I felt it was vital and highly valuable. It contributed to my experience of PPI working at its very best. It affirmed my underlying motivation and belief that what we lay researchers do make a difference |
| Travelling and committing time away from home could be a practical challenge to participation | U | There were some practicalities involved in co-research that might pose a challenge to other PPI members wishing to undertake the role of lay researcher. For example, travelling to participants’ homes and conducting the interviews meant that I had to commit time away from home |
| Expectations from researchers and a responsibility towards informants could pose pressure, for which preparation, training, and debriefing were key | U | The academic team had an underpinning expectation of us lay researchers meeting very high standards. Similarly, the research participants’ investment in the study deserved a highly skilled interviewer to enable them to openly talk about their experiences. These feelings put a lot of pressure on me. The preparation, training, and debriefing provided by the academic researcher were key in boosting my confidence to undertake the role |
| Experiential expertise could mean emotional affect | U | However, as my life-experiences paralleled those of the participants, unlike the professionals, I could not see the interview session purely as an academic process. This made co-researching such a worthwhile part of my journey, but it also affected me emotionally. Retrospectively, I think I should have asked for further support from the academic researcher to work this through.[…]These difficulties can only be overcome through considerable training, in-depth support, and trust. |
| A change of culture is still required in the academic environment at large, to embrace the added value that lay researchers may  bring to research studies | U | While these were embedded in the co-research team, I think that a change of culture is still required in the academic environment at large, to embrace the added value that lay researchers may bring to research studies. At times, I felt that my contributions in the research agenda were undermined, as they did not conform to ‘rigorous’ academic models. The lack of recognition of what true collaboration in research means made me more guarded to undertake some of the co-research tasks and presented as a challenge for me to work through personally, also at the level of professional rapport with members of the academia. |
| Participation was a powerful personal experience and enabled me to prepare for a future | U | The experience for me has been very powerful. To meet, at last, with those taking part in the study felt like truly being embedded in the research process. It gave me further insight into others’ lived experiences, enabling me to prepare for a future, which could see me on a parallel journey |
| Facilitators created the right atmosphere and fostered positive group Dynamics | U | l participants described how facilitators created the right atmosphere and fostered positive group dynamics. They spoke of the warm, receptive, down-to-earth nature of the group: ‘The people running the project are very approachable and very welcoming and they are really nice and easy to work with’ ‘The professionals are a caring group of people who are good listeners. They are a very friendly group of people who have a lot of respect for each other & obviously get on very well with each other’. |
| Researchers being reassuring, made them feel comfortable and enabled for them to open up and express views and opinions | U | Participants also appreciated the approach of the research team who provided reassurance, made them feel comfortable and valued and enabled them to express their views and opinions: ‘The researchers made us feel very comfortable and we were in a safe place and we could talk freely’ |
| Atmosphere led to participants feeling able to disclose very personal experiences that led to perceived benefits | U | This atmosphere sometimes led to participants at the workshops feeling able to disclose very personal experiences that led to perceived benefits for that individual: ‘I had kind of forgotten all about it but it was good to talk about it…because for a lot of carers you don’t get the opportunity because your friends and relatives don’t want to know and so it is probably, yea, it is a really selfish thing but actually doing this has been a really cathartic thing to me personally and it’s been great’ |
| Co-design process can be a gradual learning curve for all participants, members and researchers alike | U | Some participants reflected on the iterative nature of research and the co-design process highlighting that it can often be a gradual ‘learning curve’ for participants, members and researchers alike: ‘I think it was a bit of a learning curve, if I’m honest, for both the researchers and for the PPI because I think neither one of us knew really what were going to be our roles’ |
| A lack of diversity was noted, which was a limitation | C | While questionnaire responses indicated that most group members felt they heard a wide range of views within the group, one noted a lack of diversity: ‘Mostly older people & white British’ |
| Being listened to and make a difference was a positive experience | C | ‘As a family carer I was able to bring experience of caring for my mother who had dementia and I felt some things were changed as a result of being listened to’ ‘Comments on manual taken on board |
| Being listened to - make a difference | U | Most participants felt that they were there to genuinely influence the discussions and that their views were taken on-board by the research team: ‘You know, they are a nice team to work with, everybody was. So we weren’t just there as token…it wasn’t tokenistic or anything like that. No, I think we did bring our views and I think they were taken  on-board and I think we all…I think that was one of those where we worked quite well’ |
| They would have liked more information about the impact their involvement had made | U | Two NIDUS-family participants said that they would have liked more information about the impact their involvement had made: ‘I would have liked to be kept informed of the progress after my involvement ended’.  ‘On that basis… I’m pretty ignorant of the effect I’ve had’ |
| Wants more information in-between workshops | U | Co-design workshops were held several weeks apart and contact in between with participants varied. While most respondents indicated that they understood the purpose of NIDUS and had sufficient support and information, three family carers who participated in NIDUS-family co-design indicated that they would have liked more communication, before and between meetings: ‘Periods of silence in between meetings were too long…hard for non-academics to keep track of where we were [with the project]’ ‘I didn’t really know what was happening… You still feel some involvement in it, but it’s, sort of, been…I feel, as you say, a bit distanced from it’ |
| FC felt PPI members had less clarity than researchers about what was happening, and this could be a barrier to meaningful engagement. | U | One of these family carers felt PPI members had less clarity than researchers about what was happening, and this could be a barrier to meaningful engagement: ‘I think sometimes the research, you know, it was slightly muddled…sometimes get a bit confused about how the manual was supposed to work, at what…which point it was going to be used when…You know what I mean? Not necessarily something that we would prepare for because it was all totally clear to them. I suppose that’s partly the process…’ |
| FCs lacked opportunities to provide feedback | U | Another family carer echoed these calls for clarity around opportunities to feedback as well as information about how existing interventions might inform the NIDUS-professional manual: ‘More notice of dates of meetings if possible. At Nov(ember) meeting, I had prepared some comments on Session 1, but there did not seem to be an opportunity to provide this feedback. Since materials from (a previous study) are being used in the training programme, I’d like to know more about that’. |
| FCs requested better sense of what was happening in all streams of the programme | C | More consistent contact between meetings and regular project updates were suggested as improvements. As NIDUS is a large programme of work, participants requested a visual timeline of progress of the whole project so that they had a better sense of what was happening in all streams of the programme: ‘I think possibly it would be helpful just to have, like, a process chart, something that maybe maps out where you are in an update, even a tiny little diagram. I don’tmean something ladenwithwords or whatever, but ‘this is where we are an this is where we hope to get by…’… a visual thing’ |
| Decisions around the content of the interventions had already been made by researchers before they were consulted | U | One family carer felt that decisions around the content of the interventions had already been made by researchers before they were consulted: ‘I got the impression that I was telling them probably useful information but too late. I think they’d already made up their mind what they wanted to do and it was too late to take on-board any further even if it might have been useful’ |
| It was important that the group used a common language to communicate and translate research in an accessible and inclusive way. | U | It was important to SAC members that the group used a common language to communicate and translate research in an accessible and inclusive way. This was reflected in their preference to have scientific literature adapted by the project team into more accessible forms of information delivery, particularly by way of visual alternatives.  “[The project lead] was very cognizant that not everybody in the room had a large or small research  background. We started everything at the beginning……. Visuals. Visuals. Visuals, visuals, visuals.” |
| They appreciated when the project team would avoid the use of technical jargon and would deliver information, revisiting and reinforcing the information repeatedly over the project period. | C | Respondents reflected that not only did they prefer when information was shared in an accessible structure and format, but that they appreciated when the SAC and project team would avoid the use of technical jargon and would deliver information, revisiting and reinforcing the information repeatedly over the project period during  meetings and symposia. |
| The meeting times and locations were important. | U | The meeting times and locations were important to all members of the SAC. The community members’ needs were prioritized when choosing meeting times and locations, although the logistics were challenging to ensure all SAC members could participate in every meeting. “…but obviously [meeting] location was forever North. That’s a problem.” |
| The council benefitted from the diverse representation of professional and lived experiences among the group | C | SAC members expressed that the council benefitted from the diverse representation of professional and lived experiences among the group. They felt they brought something unique to the group from their different professional and lived experiences. |
| The project team needs to be ‘dementia aware’, including having knowledge about dementia and being respectful, patient, and considerate of the accommodations individuals living with dementia and family care partners may need to stay involved | C | Respondents felt that members of the project team responsible for facilitating the SAC activities need to be ‘dementia aware’, including having knowledge about dementia and being respectful, patient, and considerate of the accommodations individuals living with dementia and family care partners may need to stay involved. |
| Recognizing a lack of representation of minorities as a limitation | C | Recognizing a lack of representation of minorities as a limitation |
| The SAC valued when facilitators would take a transparent approach to research, compared with a predatory approach, sharing their intentions in understandable terms and inviting collaboration  of stakeholders throughout the research process | C | They perceived a typical researchers’ approach to engagement, which was often viewed as predatory, where researchers enter communities to extract information without giving back. The SAC valued when facilitators would take a transparent approach to research, sharing their intentions in understandable terms and inviting collaboration  of stakeholders throughout the research process. “[The facilitator’s] leadership style is intentional, it’s purposeful, it’s inclusive, and it’s also very humbling, which—I don’t think it’s a common word choice to use in research, but their research approach is very—confident enough to humble and be, like, ‘These are my areas of expertise. This area or this community is not.’… They seek it, they welcome it…” |
| Feeling valued, having a unique space and role was rewarding | C | Respondents across all representative stakeholder groups shared that they were valued and respected for their contributions. Having a unique space and role on the council was important to members, some reflecting a sense of pride and responsibility as representatives of a wider network of stakeholders. “It’s been a rewarding experience for me to feel like I’ve had a voice in it and, hopefully, have represented other caregivers well in my concerns and shared experience with those on the committee who haven’t been a caregiver.” |
| A transparent approach is appreciated | C | A key value shared by respondents was the cultivation of an inclusive environment where SAC members could feel comfortable to share their opinions and perspectives. Inclusivity was often attributed to the nonhierarchical structure of meetings where all members of the SAC were invited to share and listen from their unique perspectives.  “From the first day of the meeting it was very evident that that's why we were there, and everyone was sharing their thoughts and concerns, including [my spouse living with dementia]." |
| Altruistic reasons as an incentive | U | SAC members were motivated to participate in the project for different reasons, many expressed an altruistic incentive to contribute to dementia care research. They had a strong desire to help others with their work, recognizing their participation might inform the direction of future research that could benefit others. Their altruistic motivations extended from their desire to help their friends, families, as well as society at large. Being part of  something bigger was important for SAC members who recognized that not everyone had the ability to participate in similar opportunities. “I know that with that responsibility comes the benefit directly to impact my family, myself, my community, society. I’m cognizant of that, and so I happily take it with that in mind.” |
| Most SAC members identified the need for a cultural awareness in the group and in the activities of the project. | U | Most SAC members identified the need for a cultural awareness in the group and in the activities of the project. They felt it was important to seek diverse cultural representation within the council structure and to foster an environment that is inclusive for different ethnic and cultural backgrounds. Some SAC members of stakeholder groups that are often excluded from collaboration for research, including care partners and individuals from ethnic and racial minorities, felt a sense of responsibility to represent and educate others about their lived experiences. “We need to get people who look like the people that we're trying to reach: the Hispanics, the African Americans, the minorities, the people who are not as privileged. It can't be the perception of this is a white person's cure.” |
| Developed skills could translate to personal lives such as caregiving skills | C | The development of knowledge and skills through participation on the SAC was an added benefit for interviewees who felt they could translate their skills to their personal and professional lives. “Yes, definitely, the biggest part, obviously, to me is I wanted to improve—to learn about ways to improve my caregiving with fact- based, research-based techniques that have been applied, that professional – that works, or doesn’t work, or semi-works, or it  works in conjunction with A, B, C, for sure.” |
| Socialization, sharing, and listening to other people's stories are benefits | C | Further, they felt their participation provided opportunities for socialization, building their communities, and the chance to share and listen to diverse perspectives and stories. The element of peer support and social connection was particularly resonant in the context of the COVID-19 pandemic, where SAC members were experiencing the social consequences of distancing safety precautions. “I don't want to use the word “fun” but fulfilling. It's been fun. It's been supportive for me, and I believe for Michael… Just knowing that we're not isolated has been extremely helpful |
| They felt they met and exceeded the goals of the original project and had confidence that their work would make a difference in the field of dementia care research | C | SAC members expressed that they felt they met and exceeded the goals of the original project and had confidence that their work would make a difference in the field of dementia care research. Interviews reflected a sense of accomplishment and belief in the purpose of the project. "I think we accomplished a lot in that, and painstakingly, I know, for those who have been at the helm. I think a lot of useful information will be obtained for research purposes...” |
| PPI as an opportunity to collaborate and interact with medical, government staff and researchers | C | The participants acknowledged that PPI resulted in an opportunity for patients and caregivers to learn about research and become empowered by research involvement. “We often build walls between us and medical and  government staff… It is great to have a place and opportunity to collaborate and interact with them through research” |
| Participants recognized the inadequacy of systems for supporting PPI in academic fields | C | Participants recognized the inadequacy of systems for supporting PPI in academic fields, specifically, three subthemes related to issues surrounding employment of patients as researchers, leading to limited access to training courses for research skills and ethics; performance evaluation of researchers; and membership available only to academics |
| The participants in all groups were concerned about equality in the partnerships, especially given the differences in power between individuals | C | The participants in all groups were concerned about equality in the partnerships, especially given the differences in power between individuals. The second pertained to researchers' lack of PPI‐related skills for properly forming a consensus in the face of diverse opinions of patients and caregivers.“Equality is very important. I think it is crucial to have a proper discussion in a partnership, but that is the most difficult part of the current situation in Japan” |
| Expected that PPI will improve research, outcomes and dissemination | US |  |
| PPI as an opportunity to become more familiar with research | US |  |
| PPI research might require more money and time than non‐PPI research. These burdens may result in unwillingness to engage | C | The participants were concerned that PPI research might require more money and time than non‐PPI research. These burdens may result in unwillingness to engage in research collaboration among patients, caregivers and researchers |
| Tokenistic PPI research as a concern | C | The participants recognized that there are excessive expectations regarding PPI. First, there were concerns that research without PPI and patientdriven research are not sufficiently promoted, which increases tokenistic PPI research. Second, PPI alone is not always sufficient for solving problems with no clear biological indicators of mental illness or for promoting social inclusion. Third, PPI may have less social benefit than expected, particularly in Japanese culture. “I don't feel that the research actually makes a change that will affect our lives” |
| Unwillingness to cooperate due to concerns about stigmatization by researchers | US |  |
| Explaining and discussing the entire research process with patients and caregivers using familiar language | C | Explaining and discussing the entire research process with patients and caregivers using familiar language |
| Establishing an atmosphere that allows for free, safe and active exchange of ideas | C | Establishing an atmosphere that allows for free, safe and active exchange of ideas; “I feel that it is vital to create an atmosphere where patients can talk honestly. Unless they establish such an atmosphere, researchers will not hear patients' true voices” |
| No rules regarding compensation | US |  |
| No access to being co-author | US |  |
| No access to papers | US |  |
| Too many research skills are required for new PPIs | US |  |
| Practical difficulties in implementing PPI for some diseases | US |  |
| Concerns about representativeness | US |  |
| Concerns about participants will bias opinions | US |  |
| Issues related to the feasibility of a partnership based on “real” equal relationships | US |  |
| Using plain language as a strategy to facilitate mutual understanding | US |  |
| Enthusiastically communicate about PPI as a strategy to facilitate mutual understanding | US |  |
| Explanation of terminology as a strategy to facilitate mutual understanding | US |  |
| Explanation of expected roles as a strategy to facilitate mutual understanding | US |  |
| Sharing the roles of each stakeholder group based on an understanding of the differences in individual viewpoints as a strategy to facilitate mutual understanding | US |  |
| Explanations of how to share the results of PPI studies as a strategy to facilitate mutual understanding | US |  |
| Creating an atmosphere where participants can freely express their opinions as a strategy to facilitate mutual understanding | US |  |
| Advance preparation for active exchange of opinions | US |  |
| For active exchange of opinions, ensuring opportunities to talk with people who have the same background | US |  |
| Develop a system in which PPIs are highly valued as a way to match divers objectives | US |  |
| Develop guidelines in conjunction with patient and caregivers | US |  |
| Develop training in research | US |  |
| FC should be regarded as important team members | C | Co-production involves treating family carers as equal partners in a research project. Family carers should be regarded as important team members whose voices are respected, rather than as an optional add-on. Working together as peers should be reflected in a variety of ways, including appropriate credit and authorship (where family carers want this), involving family carers in senior positions and resolving disagreements fairly |
| Co-production should be meaningful and have an impact | C | Co-production should ideally be embedded from the early stages of a research project to allow family carers to shape key decisions. It must be more than a tokenistic, tick-box exercise and requires authentically valuing family carers’ views such that they meaningfully influence the course of research. Meaningful involvement can bring enormous practical and personal benefits to researchers and family carers and so, should be mutually rewarding to all involved |
| Contributions should be welcomed with openness and curiosity, including where these are critical | C | Researchers and family carers should welcome others’ contributions with openness and curiosity, including where these are critical. Engaging with critiques can be particularly valuable opportunities for co-production to constructively address issues with a research project. This does not mean that the views of either family carers or researchers should outweigh those of others. Rather, it means being willing to engage in open dialogue and to have clear and transparent procedures for resolving disagreements |
| Clear communication is essential | C | Clear communication is essential for successful co-production with family carers. Family carers will have varying amounts of previous involvement in research and are frequently balancing many competing commitments and responsibilities. It is therefore important that researchers provide them with clear information about their role, the time commitment required and deadlines. We recommend preparing clear and mutually agreed outlines of family carers’ roles and who they can approach for support |
| Keep communication free from jargon | C | It is also crucial that researchers keep communication free from academic jargon and communicate through channels that suit family carers |
| Communicate through channels that suit family carers | C | It is also crucial that researchers keep communication free from academic jargon and communicate through channels that suit family carers |
| FG should be recompensed fairly | C | Family carers should be recompensed fairly for the time and expertise they provide. Payment should also be prompt, account for all work and out-of-pocket expenses, include replacement care costs where this is required and offer different options for how payment is made. Family carers should also be reminded that payment, including vouchers, may have implications for any welfare and tax payments |
| Be mindful of the FC well-being | C | Researchers must be mindful of the well-being of family carers throughout their collaboration. Co-production will often involve discussion of topics that are deeply personal, emotive or potentially distressing to family carers and appropriate support should therefore be in place. This includes emphasising that family carers may share as much or as little as they wish, interacting with family carers empathetically and offering debriefs or follow-up support |
| Consider that FC often are “time-poor” and have to respond to unexpected, last minute demands | C | Flexibility is critical to making co-production accessible to many family carers. Many family carers are “time-poor” and may have to respond to unexpected, last-minute demands. Researchers can therefore facilitate involvement through offering different input methods, catch-up sessions and varying degrees of involvement for family carers with different amounts of time. Different family carers may ultimately need different things, and researchers should be willing to explore reasonable adaptations that can be made to allow people to contribute |
| We’re kind of living and breathing what we’re actually advising people to do. So it should have been, and I believe it was, a really exemplary piece of co- production | C | It’s quite strange, a strange thing we’re kind of living and breathing what we’re actually advising people to do. So it should have been, and I believe it was, a really exemplary piece of co- production |
| Synthesising many different perspectives is a challenge | C | I think what was challenging [...] is that there’s so many voices and perspectives, and we didn’t necessarily know exactly what the piece of work we were producing was going to end up looking like. So the challenge was navigating our way through that while kind of keeping the faith that we were going to end up with something meaningful and useful. |
| Lack of diversity | C | I also hope that somehow we will reach a really diverse selection of parent carers. I think we really need that diversity across all aspects of diversity, and make sure that more seldom heard voices are heard |
| Group work was viewed as peer-support | C | Participants discussed the benefits of group work. Group work was viewed as peer support for participants, which was seen as a valuable way to help understand each other’s circumstances and needs to support each other |
| Research awareness training facilitated contribution | C | Participants reported that they had gained new knowledge and discussed the benefits of receiving the RAT for the role, highlighting that the structuring of the training alongside the involvement tasks as factors that helped them to contribute to research |
| A human relationship with researchers made them feel they were making a valuable contribution | U | RUG participants also reported that the support provided by the local PPI coordinators made them feel that they were making a valuable contribution. A participant commented on the importance of the ‘human relationship’ (Athens, RUG4, care partner) with the coordinator |
| Interaction with researchers and having researchers listen to RUG member’s opinions to was reported as an important aspect. | U | Interaction with researchers and having researchers listen to RUG member’s opinions to was reported as an important aspect. “We said something and they ‘listened’ to it and we move forward, they tell you a little more, something like that …. Yes, for sure. Otherwise, I wouldn’t be here today. It’s a good feeling, to be heard. You gain this feeling that you are not alone and that there are people that care for you, and that what you say can go further” |
| Receiving feedback on that contribution was having a tangible impact was important | U | Receiving feedback was also highlighted as an important factor in letting RUG members know that their contribution was having a tangible impact: I feel that our communication was quite satisfactory and apart from that we had feedback |
| Personal benefits were the opportunity to share issues with others in similar position, knowledge, interacting with others, giving advice and making contribution | C | Face-to-face group meetings provided RUG members the opportunity to share issues with others in similar position. RUG members reported that the experience personally benefited them in terms of gaining new knowledge, interacting with others, giving advice and feeling that they were making a worthwhile contribution. RUG participants felt their involvement exceeded their expectations, due to continuous involvement and training in research awarenes. |
| Their perspective provided researchers with a better understanding of the impact of dementia that informed research decisions | U | RUG members talked about how providing their perspective provided researchers with a better understanding of the impact of dementia that informed research decisions. RUG members emphasised their contribution in making written materials more accessible for participants and suggesting changes in the interventions and tools developed by the research team. RUG members reported that incremental changes informed by PPI could make a difference to practical outcomes: “Well it’s just one factor in the complicated chain of conditions that lead to the outcome. So it’s not going to have any direct impact on any individual people, but every time a member of the group makes a contribution, it’s adding a little bit more information to the whole package of information that’s going to improve the lives of the end users in the long term” |
| Difficult to take on responsibilities over and above caregiving | U | Caregivers felt “wired” into their roles and found it difficult to take on responsibilities over and above keeping the health of their loved one stable |
| Turn lived experience to something positive | C | I was always on the lookout for changing things, making things better. I just feel I’ve got so much experience, so much knowledge, and so much sadness, I have to turn it into something positive. I need something good to come out of this. So that’s always been my driving force. I have to use it somehow |
| Proximity facilitates involvement | C | I do a lot of advocacies on Capitol Hill, again because of my proximity, being an hour  away there’s no excuse not to. (Caregiver, United States) |
| Some initially struggled with their role because they viewed themselves “on a much lower level” than other members of the research team | C | Some participants felt valued and integrated in collaborative research teams, whereas others  described a chasm between “us patients/caregivers) and them (clinicians/researchers)” and experienced “a real pushback against treating the patient as an equitable partner.” Some initially struggled with their role because they viewed themselves “on a much lower level” than other members of the research team (i.e., clinicians). |
| With encouragement came confidence | C | With encouragement from the researchers, they developed the confidence to “speak up.” |
| Responsibility to End Users | C | As lone voices in research groups, participants reminded the team of “why they’re doing this research” by sharing firsthand insights of their lived experience. Participants considered themselves as advocates for other patients/caregivers and felt a responsibility to use the opportunities they were given to represent the broader patient voice, particularly for specific groups that were often underserved |
| Research as an opportunity to gain input from others | C | Research was seen as a needed opportunity to gain input from others: “there’s no way it wouldn’t help people, because everybody has different ideas, and no one knows everything” |
| Research could provide strategies to deal with stress or improve care | C | Caregivers shared that research could provide strategies to help them deal with stress or improve the care of their loved ones: “[Research] could help tell us some ways to help deal with the stress, ways to help with different ailments that affect our family members” |
| Altruistic reasons for participating | C | “me teaching someone else or helping them learn ways to do better at your caregiving would make me feel so good” |
| Gaining knowledge as a motivator | C | Participants also discussed gaining knowledge as a motivator: “I’m always willing to learn a new field. Anything that gives me more knowledge” |
| Social connections as a motivator | C | Other motivators included forming social connections, keeping busy, feeling useful, and participating in activities they enjoy, such as “I like to analyze stuff” |
| Just being able to have a discussion with other people who go through what I go through, so I don’t feel like, half the time, I’m going crazy | C | Few mentioned money as a potential motivation, such as this caregiver: “Just being able to have a discussion with other people who go through what I go through, so I don’t feel like, half the time, I’m going crazy. I don’t think you have to pay people to do research. I think people want to do research so they can find a better outcome. I mean, some  people want to get paid, I guess, but that would just limit your ability to learn things” |
| Lack of social skills as a barrier | C | Some participants felt they had insufficient social skills: “I’m not good with people” |
| Insufficient knowledge as a barrier | C | Other participants felt they had insufficient knowledge: “I’m no expert” |
| Outside perspective as a contribution | C | One caregiver seemed reluctant about the value of their contributions, stating “I’m not sure what layman would be able to help with on that, but I guess I could help get an outside perspective” |
| Caregiving responsibilities as a barrier | U | For caregivers, time constraints caused by work or caregiving responsibilities were common, such as: “I have to stay home with Mom” |
| Participating as an advisor would disrupt their daily routine | U | Some participants felt that participating as an advisor would disrupt their daily routine … For example, one caregiver stated: “We just like to live a quiet life here and try to make the best of everything goes on with us here … and nothing else matters to us that much anymore, really” (s1, caregiver). For these participants, participating  as a research advisor would take time away from valued activities: “I kind of want to be outside enjoying outside or talking to a friend or something where I don’t have to think about anything else” (s2, caregiver). |
| Importance of flexibility | U | As one caregiver pointed out, the advantage of videoconferencing is that “if we did it on video, it’s like meeting in person. And people’s schedules are weird, so you have to be flexible” |
| Exchange of experiences as a motive | U | I have expe­riences that might be valuable, but other people on the council may have experiences that could help me as well” |
| Reciprocal learning as a motive | C | The family advisor expressed that engag­ing with health professionals on the research team (e.g. nurses, physicians, social workers) allowed for reciprocal learning, where the care partner learned from the health professional and the health professional had the chance to learn from caregivers |
| Opportunity to learn about interventions and programs | C | Opportunity to learn about interventions and programs that are happening in other countries |
| It allowed family advisors to be active in care | U | it also allowed family advisors to be active participants in their loved one’s care and not “only visitors” |
| A desire to create a circle of moral and psychosocial support system for other carers. | U | Based on their own experiences, fam­ily advisors expressed a strong desire to create a circle of moral and psychosocial support system for other carers. Advisors endorsed that their personal experience with a loved one gave them “the drive to do something” |
| Feelings of frustration sparked something within, which led to participation | U | The advisor further attributed that the lack of information from the physi­cian led to feelings of frustration but then ‘sparked some­thing within’, which led the participant to determine that: “rather than fighting the system, I educated myself. I just decided I would speak out and tell mom’s story to change those mistakes and hopefully make a difference” |
| To help others navigate the health system | C | A strong motivating factor that led advisors to engage with the Strategic Guiding Council and research team was to help others navigate the health system |
| Use their voice to improve the qual­ity of care as motivation | C | Family advisors are motivated to be engaged with the Strategic Guiding Council to be able to use their voice to improve the qual­ity of care provided to individuals with dementia and use their voices to “support or influence the direction of the program |
| Co-designers wanting to contribute to something worthwhile | C | With co-designers wanting to contribute to something worthwhile and to advocate for individuals with aphasia. |
| Interest in improving technology for people with aphasia | U | A specific interest in improving technology for people with aphasia: ‘I found the tools that exist now are really wanting. They fall short of the mark. So I was really motivated to find better options… I’m excited about doing this technology part of it’ |
| Time constraints and lack of knowledge as barriers | C | time commitments and feeling hesitant about being knowledgeable enough as potential barriers. Following co-design workshops, time commitment was no longer identified as a barrier. Not being knowledgeable enough was also no longer identified as a barrier |
| Illnesses and life events as interfering | C | Other personal factors (illness/health and life events) interfered at various time points for some individuals |
| Resources were perceived as both barriers and facilitators | C | Resources were perceived as both barriers and facilitators. For example, although one co-designer found the prompt cards useful for prompting and grounding ideas, others believed there were too many cards, making them confusing and challenging to incorporate. Some co-designers found the workbooks helpful, but others found them too detailed, vague or time-consuming. |
| Information provision was perceived as a facilitator | C | Overall, information provision, including workshop content, was perceived as a facilitator, with co-designers describing information as ‘clear and concise’, and reporting information/instructions were well sign- posted |
| Enjoyment of the belief that co- designer ideas had been listened to and included | U | Co-designers’ comments highlighted enjoyment of the collaborative nature of co-design, and the belief that co- designer ideas had been listened to and included: ‘Just watching the contribution of people living with aphasia too, it was terrific. I feel like they took on board our suggestions and incorporated them into the product’ |
| Working towards a common goal was positive | C | Positive about working towards a common goal. A high level of connectedness was reported |
| Co-design as valuing diverse perspectives | C | There was an expectation that co-design groups value diverse perspectives and lived- experience knowledge. Post-interview data indicated these expectations had been met |
| Groups as an inclusive and safe place | C | Groups were also described as inclusive and a safe place to share all ideas |
| Researchers' communication style, planning and organisation and inclusiveness were appreciated | U | Co-designers gave positive feedback about the research team with respect to communication style/strategies, planning and organisation, and inclusiveness/openness to ideas: ‘The whole team was listening and they weren’t trying to push their own barrel of expertise’ |
| Social connectedness as a positive outcome | C | Four co-designers reported feeling more socially connected as a result of building friendships and professional connections within the co-designer group |
| Positive impact on the relationship with the patient as a positive outcome | U | one family member reported an unexpected positive impact on her relationship with her spouse with aphasia who had also participated: ‘That was an amazing plus for us, because we could… talk about those kinds of things together and really have that double involvement. It’s like one and one makes 11… I think we got a heck of a lot out of this for us’ |
| Contributing to society by sharing  their experience was identified as beneficial | C | Contributing to society by sharing their experience was identified as beneficial |
| The partnership's reciprocal nature was beneficial | C | Contributing to society by sharing their experience was identified as beneficial, so was the partnership's reciprocal nature. That is, the researcher and `Research Buddies` learnt from each other. Specifically, the‘embedded consultation’ approach allowed the researcher to explore issues important to family carers and gain insights into the booklet's implementation from a family carer perspective, which could not have been gleaned from supervisors or academic resources |
| Sharing the pitfalls and successes of the research was a positive experience | C | Sharing the pitfalls and successes of the research was a positive experience for both the researcher and the Research Buddies |
| EBCD/AEBCD provided former patients  and carers with the means and confidence to give voice to their experiences | C | EBCD/AEBCD provided former patients and carers with the means and confidence to give voice to their experiences, and appeared to help these participants understand how they could work with staff as partners in bringing about improvements. ”We felt able to say what we wanted to say and what we wanted to say has turned out to have a valuable effect, so, yeah, very happy to say that we didn’t feel intimidated in any way.” |
| EBCD/AEBCD participants shared a common experience with peers and staff as something positive | C | Participants in EBCD/AEBCD shared a common experience with their peers and with the staff with whom they exchanged ideas and problem-solved in co-design groups. |
| Trigger films were critical in all staff reorientating their focus to patients’ experiences | C | Patients’ voices and the authentic examples depicted in trigger films were critical in all staff reorientating their focus to patients’ experiences. These events contributed to participants’ progression from shared understanding of the importance of change to working together to define ways to implement ideas into existing working practices |

FC = Family caregivers, U = Unequivocal, C = Credible, US = Unsupported, EBCD = Exerience Based Co-design, AEBCD = Accelerated Exerience Based Co-design
